# Author Correction: Prominent misinformation interventions reduce misperceptions but increase scepticism

**DOI:** 10.1038/s41562-024-01958-w

**Published:** 2024-07-19

**Authors:** Emma Hoes, Brian Aitken, Jingwen Zhang, Tomasz Gackowski, Magdalena Wojcieszak

**Affiliations:** 1 Department of Political Science, University of Zurich, Zurich, Switzerland; 2 Huron Consulting Group, Chicago, IL USA; 3 Department of Communication, University of California, Davis, Davis, CA USA; 4 Department of Communication and Public Relations, University of Warsaw, Warsaw, Poland

**Keywords:** Politics and international relations, Cultural and media studies, Science, technology and society

Correction to: *Nature Human Behaviour* 10.1038/s41562-024-01884-x, published online 10 June 2024.

In the version of the article originally published, we declared no competing interests. We have now amended our declaration to disclose funding by Facebook/Meta (previously only noted in the Acknowledgements) and a commercial affiliation for one of the authors (B.A.). These corrections have been made to the HTML and PDF versions of the article.

