# Supplementary material for: Prominent misinformation interventions reduce misperceptions but increase scepticism
Source: Nat Hum Behav. 2024 Jun 10;8(8):1545–53. doi: 10.1038/s41562-024-01884-x (PMC11343704; doi:10.1038/s41562-024-01884-x)
Supplement: Supplementary file 1 — Supplementary Figs. 1–27 and Tables 1–91. [file 41562_2024_1884_MOESM1_ESM.pdf]

nature human behaviour

Supplementary information

https://doi.org/10.1038/s41562-024-01884-x

Prominent misinformation interventions reduce misperceptions but increase scepticism

In the format provided by the authors and unedited

nature human behaviour

Supplementary information https://doi.org/10.1038/s41562-024-01884-x

Prominent misinformation interventions reduce misperceptions but increase scepticism

In the format provided by the authors and unedited

Supplemental Information

Contents

References **18**

A Stimulus Materials **23**

A.1 Fact-checking . . . 23

A.1.1 US . . . 24

A.1.2 Poland . . . 29

A.1.3 Hong Kong . . . 34

A.2 Media Literacy . . . 38

A.2.1 US . . . 40

A.2.2 Poland . . . 42

A.2.3 Hong Kong . . . 44

A.3 Coverage of Misinformation . . . 46

A.3.1 US . . . 47

A.3.2 Poland . . . 49

A.3.3 Hong Kong . . . 51

B Survey Measures **53**

B.1 Control Variables . . . 53

B.2 Outcome Variables . . . 53

B.2.1 Statements US . . . 54

B.2.2 Statements Poland . . . 54

B.2.3 Statements Hong Kong . . . 54

B.3 Consent and Debriefing Form . . . 55

B.3.1 Consent Form . . . 55

B.3.2 Debriefing Form . . . 55

B.4 Indices . . . 56

B.5 Manipulation Check . . . 56

B.5.1 Demographic Comparisons between Compliers and Non-Compliers t-test Results . 57

C Regression Tables **58**

C.1 Main Results . . . 58

C.1.1 Main Results - United States . . . 59

C.1.2 Main Results - Poland 77
C.1.3 Main Results - Hong Kong 95
C.2 Robustness Checks 112
C.3 Results for other pre-registered outcomes 121
C.4 Truth Discernment 141
C.5 Bayes Factor Analyses 144

A Stimulus Materials

The stimulus materials (the mock Facebook posts) in this document are redacted due to legal reasons. All original materials can be found on OSF at https://osf.io/5xc7k/.

A.1 Fact-checking

We manipulated exposure to the accountability fact-checking strategy by asking participants to look at two mock Facebook posts containing a variation of the following text:

- Post 1: Recently, Republican/Democrat [ACTOR] claimed that [FALSE STATEMENT]. [ACTOR]s claim, however, has been rated false by independent fact-checkers. The claim turns out to be fabricated/made up.
- Post 2: Even though Republican/Democrat [ACTOR] said that [STATEMENT], there is no evidence suggesting that this is true. In fact, a recent check by independent fact-checkers discovered that the [ACTORS] claim that [STATEMENT] is completely false.

We manipulated exposure to the correctability fact-checking strategy by asking participants to look at two mock Facebook posts containing a variation of the following text:

- Post 1: Recently, a claim was made that [STATEMENT]. This claim, however, has been rated false by independent fact-checkers. The claim turns out to be fabricated/made up.
- Post 2: Even though it was said that [STATEMENT], there is no evidence suggesting that this is true. In fact, a recent check by independent fact-checkers discovered that the [STATEMENT] is completely false.

Variation between Republican and Democrat actors was random such that each participant is equally likely to see the treatment text with a Republican or Democrat actor. The order of the posts (top/bottom) was also randomized. We carried out a pilot study in which we tested six made-up statements. From these six, we picked two statements that were rated as similarly easy to read, interesting, easy to understand, likely to be true, and believable, and therefore all equally plausible among Democrats and Republicans.

A.1.1 US

Figure A1: Supplementary Figure 1: Accountability Fact Checking - 1

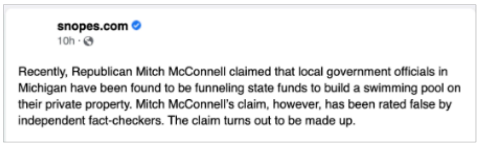

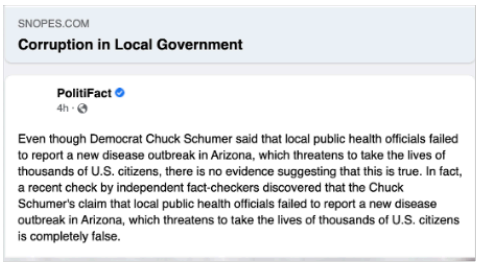

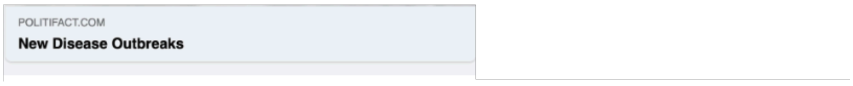

Figure A2: Supplementary Figure 2: Accountability Fact Checking - 2 - Figure 2

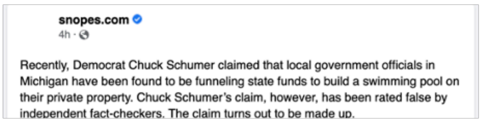

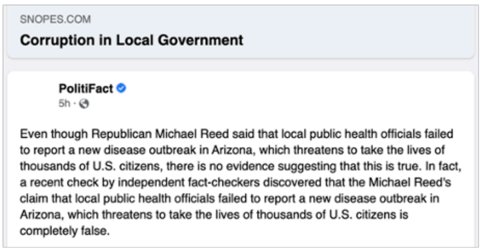

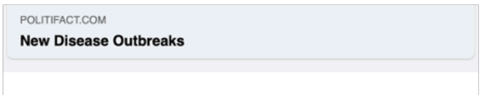

Figure A3: Supplementary Figure 3: Accountability Fact Checking - 3

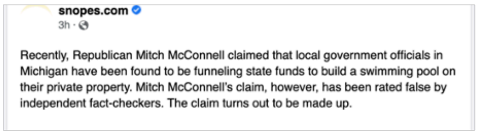

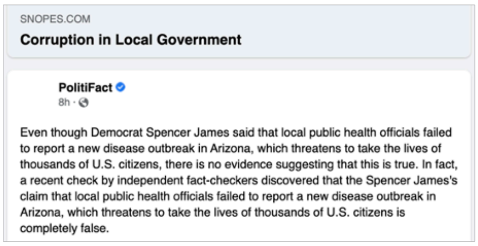

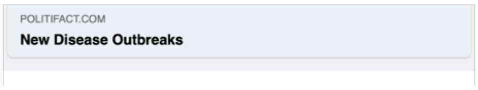

Figure A4: Supplementary Figure 4: Accountability Fact Checking - 4

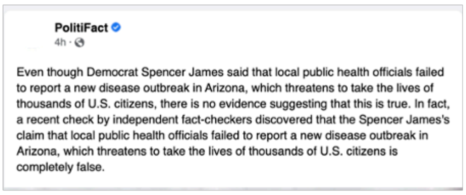
PolitiFact
4h ·

Even though Democrat Spencer James said that local public health officials failed to report a new disease outbreak in Arizona, which threatens to take the lives of thousands of U.S. citizens, there is no evidence suggesting that this is true. In fact, a recent check by independent fact-checkers discovered that the Spencer James's claim that local public health officials failed to report a new disease outbreak in Arizona, which threatens to take the lives of thousands of U.S. citizens is completely false.

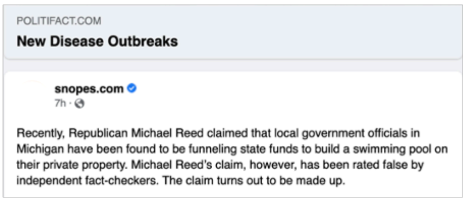
POLITIFACT.COM
New Disease Outbreaks

snopes.com
7h ·

Recently, Republican Michael Reed claimed that local government officials in Michigan have been found to be funneling state funds to build a swimming pool on their private property. Michael Reed's claim, however, has been rated false by independent fact-checkers. The claim turns out to be made up.

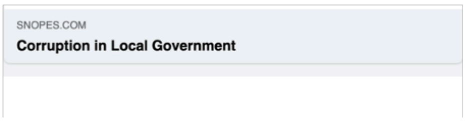
SNOPES.COM
Corruption in Local Government

Figure A5: Supplementary Figure 5: Correctability Fact Checking

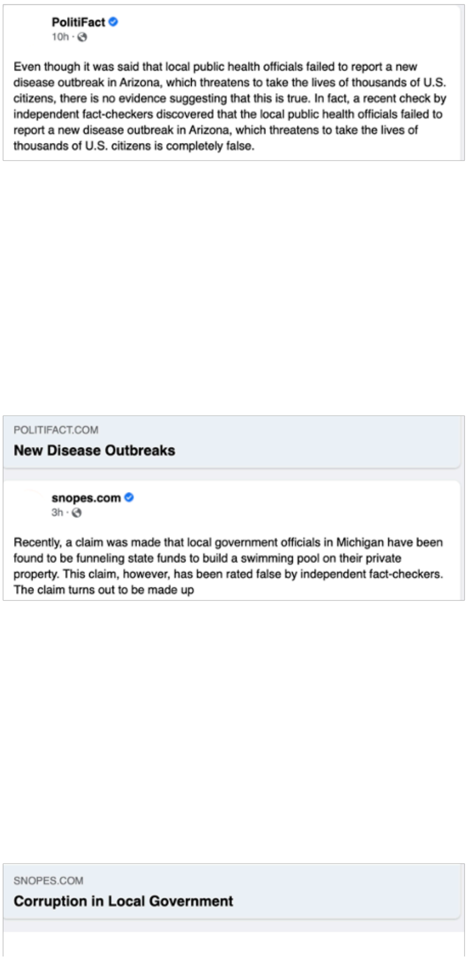

A.1.2 Poland

Figure A6: Supplementary Figure 6: Accountability Fact Checking - 1

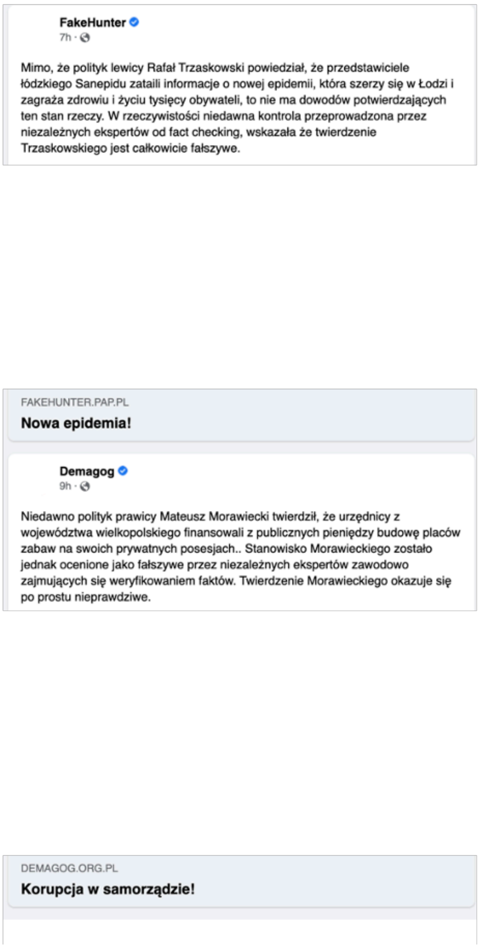

Figure A7: Supplementary Figure 7: Accountability Fact Checking - 2

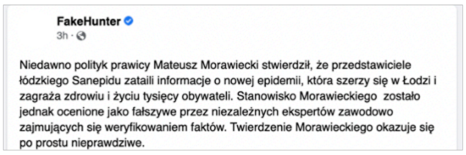

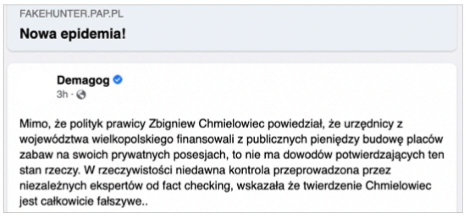

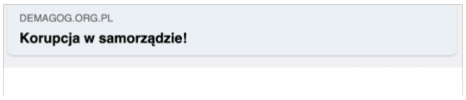

Figure A8: Supplementary Figure 8: Accountability Fact Checking - 3

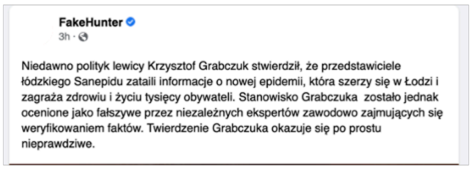

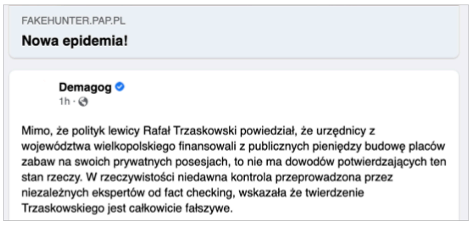

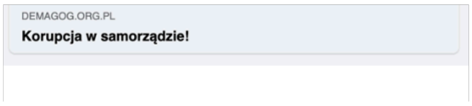

Figure A9: Supplementary Figure 9: Accountability Fact Checking - 4

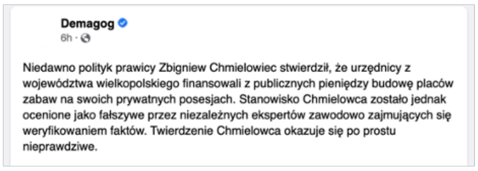
Demagog
6h ·

Niedawno polityk prawicy Zbigniew Chmielowiec stwierdził, że urzędnicy z województwa wielkopolskiego finansowali z publicznych pieniędzy budowę placów zabaw na swoich prywatnych posesjach. Stanowisko Chmielowca zostało jednak ocenione jako fałszywe przez niezależnych ekspertów zawodowo zajmujących się weryfikowaniem faktów. Twierdzenie Chmielowca okazuje się po prostu nieprawdziwe.

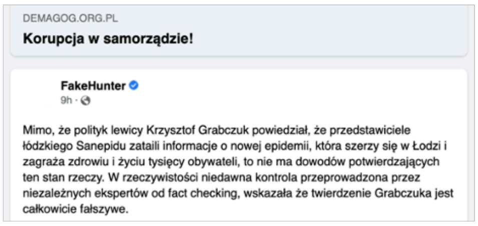
DEMAGOG.ORG.PL
Korupcja w samorządzie!

FakeHunter
9h ·

Mimo, że polityk lewicy Krzysztof Grabczuk powiedział, że przedstawiciele łódzkiego Sanepidu zataili informacje o nowej epidemii, która szerzy się w Łodzi i zagraża zdrowiu i życiu tysięcy obywateli, to nie ma dowodów potwierdzających ten stan rzeczy. W rzeczywistości niedawna kontrola przeprowadzona przez niezależnych ekspertów od fact checking, wskazała że twierdzenie Grabczuka jest całkowicie fałszywe.

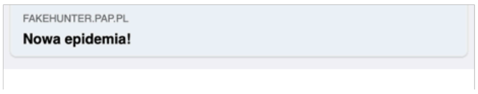
FAKEHUNTER.PAP.PL
Nowa epidemia!

Figure A10: Supplementary Figure 10: Correctability Fact Checking

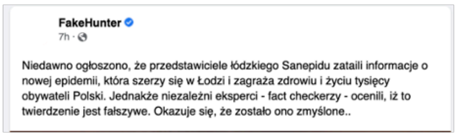

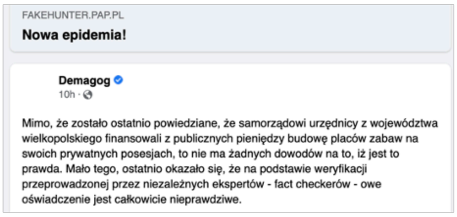

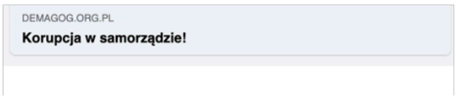

A.1.3 Hong Kong

Figure A11: Supplementary Figure 11: Accountability Fact Checking - 1

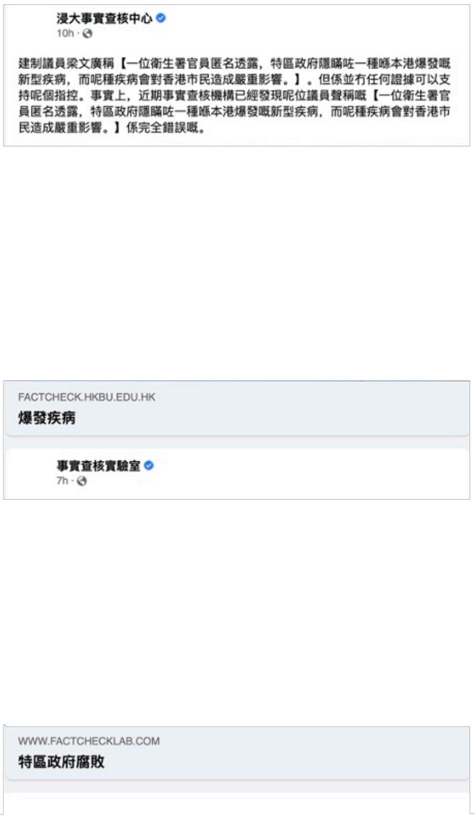

Figure A12: Supplementary Figure 12: Accountability Fact Checking - 2

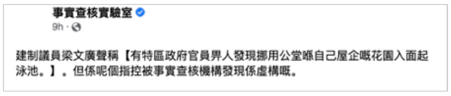

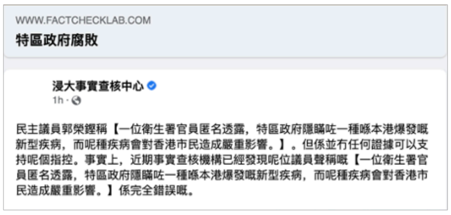

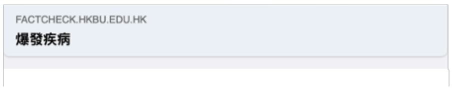

Figure A13: Supplementary Figure 13: Accountability Fact Checking - 3

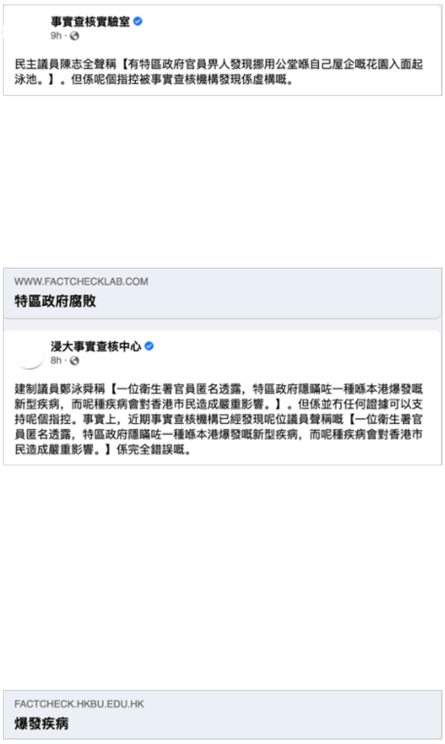
事實查核實驗室
9h ·

民主議員陳志全聲稱【有特區政府官員畀人發現挪用公堂喺自己屋企嘅花園入面起泳池。】。但係呢個指控被事實查核機構發現係虛構嘅。

WWW.FACTCHECKLAB.COM
特區政府腐敗

浸大事實查核中心
8h ·

建制議員鄭泳舜稱【一位衛生署官員匿名透露，特區政府隱瞞咗一種喺本港爆發嘅新型疾病，而呢種疾病會對香港市民造成嚴重影響。】。但係並冇任何證據可以支持呢個指控。事實上，近期事實查核機構已經發現呢位議員聲稱嘅【一位衛生署官員匿名透露，特區政府隱瞞咗一種喺本港爆發嘅新型疾病，而呢種疾病會對香港市民造成嚴重影響。】係完全錯誤嘅。

FACTCHECK.HKBU.EDU.HK
爆發疾病

Figure A14: Supplementary Figure 14: Accountability Fact Checking - 4

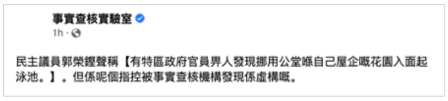

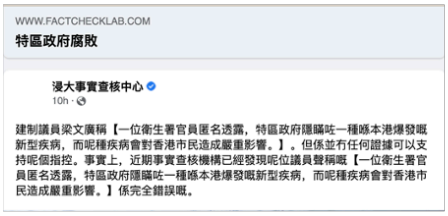

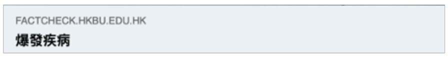

Figure A15: Supplementary Figure 15: Correctability Fact Checking

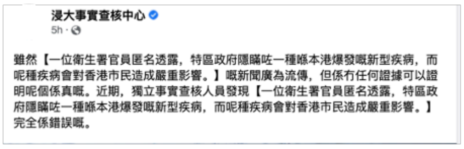

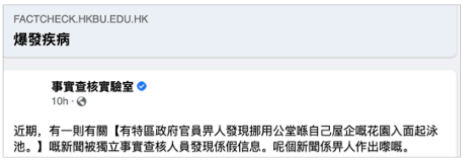

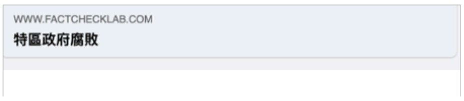

A.2 Media Literacy

We manipulated exposure to misinformation focus as a media literacy strategy by asking participants to look at two mock Facebook posts containing a variation of the following texts (see Appendix B for the text accompanied by icons):

- Post 1: **Is the source fake?** Click away from the story to investigate the site, its trustworthiness and contact info.

  Consult fact-checkers. To check if the story is false, consult fact-checkers to be sure what you are reading is true

  Is it a joke? Check whether the source is known for parody. False news stories and parody may look alike

  Is the headline misleading? False news stories often have shocking, unbelievable claims in the headline

- Post 2: **Is the author legitimate?** Do a quick search on the author to determine if the author is faking to be a journalist or expert

Is it timely? Fake news stories are often reposted old content that are not relevant or misattributed to todays events

Credible sources? Check if the links lead to credible sources. Do the links work?

Check your biases Understand your own beliefs in order to avoid falling for fake news articles that are in line with these beliefs

We manipulated exposure to the bias focus as a media literacy strategy by asking participants to look at two mock Facebook posts containing a variation of the text below (see Appendix B for the text accompanied by icons). We aimed to stay as close to the original infographic as much as possible. The infographic was originally published by the International Federation of Library Associations and Institutions (IFLA), in 2017, and has been spread on social media widely and translated into multiple languages. We slightly amended some of the texts on the original infographic so that the Bias Focus and Misinformation Focus would at the very least have a similar layout and tone, while isolating/manipulating specific differences between the treatments which could potentially be driving any effects.

- Post 1: **Is the source biased** Click away from the story to investigate the site, its political leaning, and the about page

  Consult others. To avoid falling for very biased news that are in line with your beliefs, understand your own beliefs and consult multiple sources

  Is it mockery? Check whether the source is known for mocking some political or social groups. Some news outlets are meant to derogate others more than to inform

  Is the headline biased? Often headlines indicate the political bias of the news story and help spot biased news

- Post 2: **Is the author biased?** Do a quick search to verify whether the author is likely to favor one political party, viewpoint, or group

  Is it relevant? Some biased articles reiterate events from the past for political gain. Check to see when this event occurred and if it is relevant to today.

  Biased supporting sources? Are the sources cited from one political party only or are both political sides represented?

  Reflect on your biases News organizations tailor articles towards specific audiences. Understand how your own biases might be playing a role in what you think is objective.

A.2.1 US

Figure A16: Supplementary Figure 16: Bias Focus Media Literacy

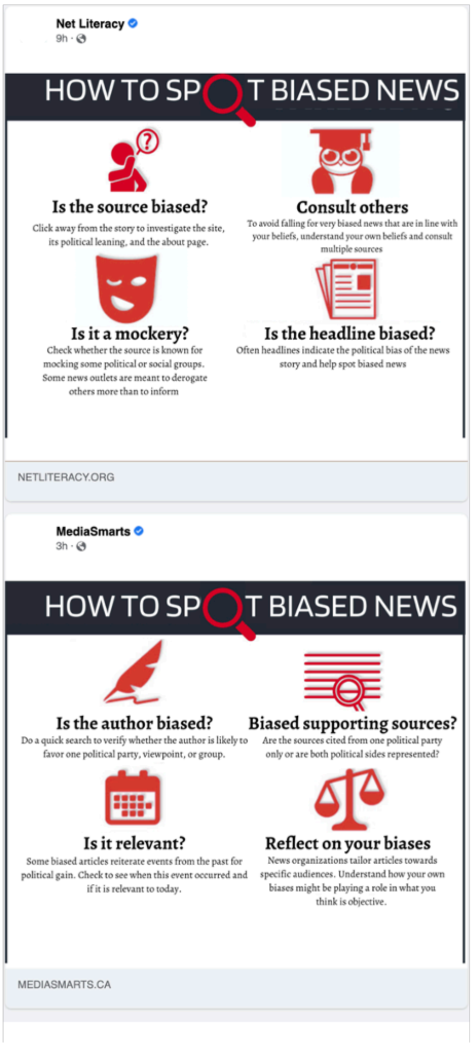

Figure A17: Supplementary Figure 17: Misinformation Focus Media Literacy

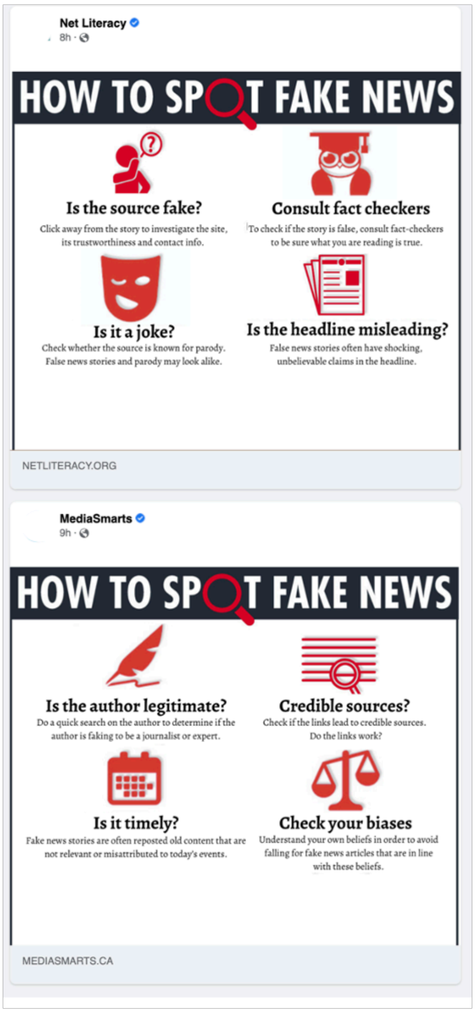

A.2.2 Poland

Figure A18: Supplementary Figure 18: Bias Focus Media Literacy

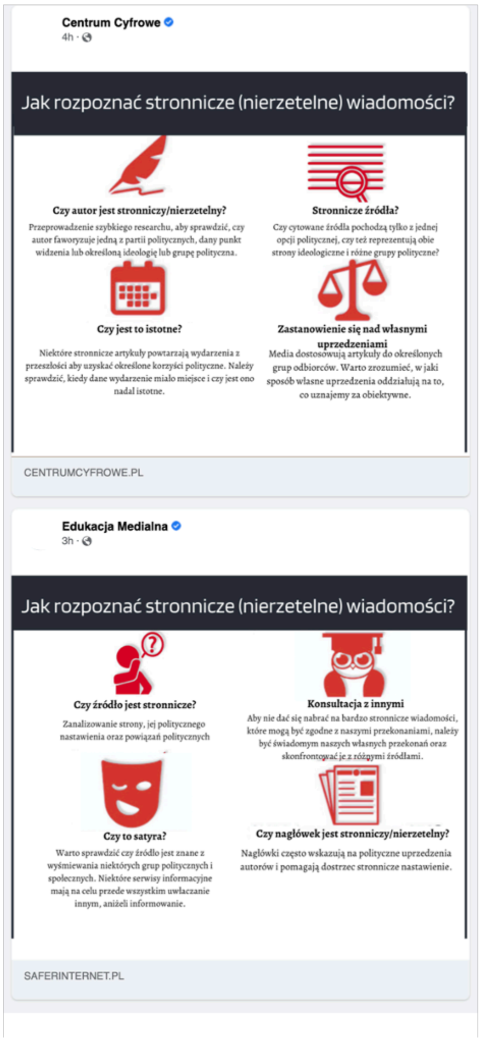

Figure A19: Supplementary Figure 19: Misinformation Focus Media Literacy

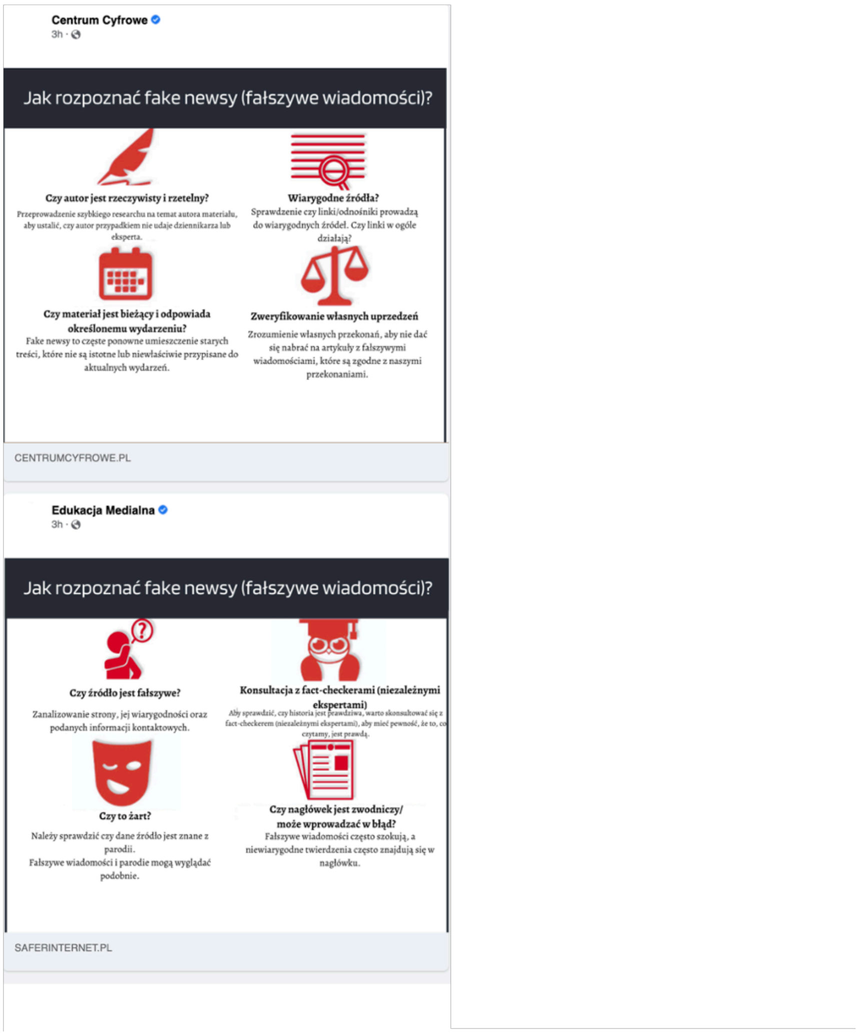

A.2.3 Hong Kong

Figure A20: Supplementary Figure 20: Bias Focus Media Literacy

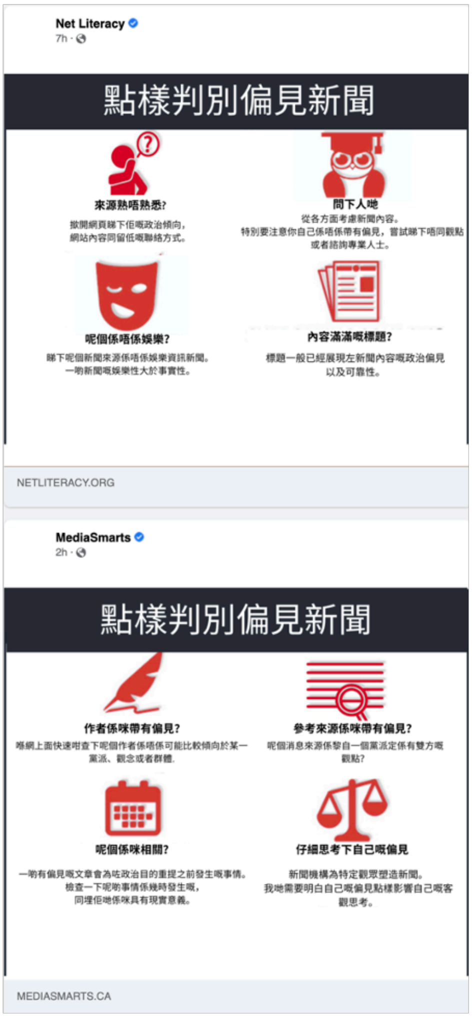

Figure A21: Supplementary Figure 21: Misinformation Focus Media Literacy

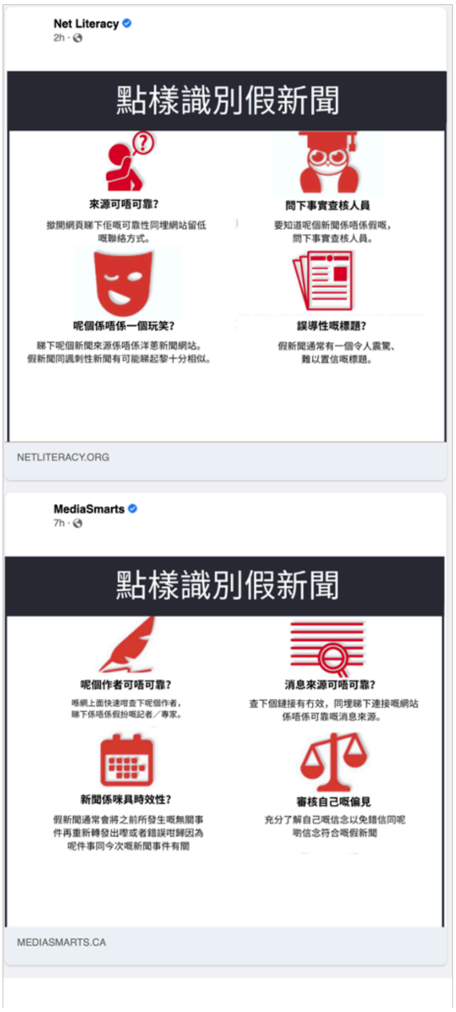

A.3 Coverage of Misinformation

We manipulated exposure to the de-contextualized approach by asking participants to look at two mock Facebook posts containing a variation of the following texts (see Appendix B for the stimuli material):

- Post 1: A lot of information about [TOPIC] is false and is causing an infodemic. The false claim that [STATEMENT] has been circulated widely. A growing number of people are trying to fight fake news by stopping the spread of such false claims.
- Post 2: There are many efforts trying to stop the spread of fake news about [TOPIC]. Despite these efforts, false [STATEMENT] and other fake news are still circulated online. The number of fake news is still worrying many.

We manipulated exposure to the contextualized approach by asking participants to look at two mock Facebook posts containing a variation of the following texts:

- Post 1: Despite concerns that there is a lot of misinformation about [TOPIC]), research shows that only few people encounter or share fake news such as [STATEMENT]. A growing number of scholars are trying to make people aware that fake news is not as prevalent as they think.
- Post 2: Many people worry that misinformation about [TOPIC] is widespread. But recent studies find that most Americans never see any fake news, such as [STATEMENT] We need to increase the efforts to educate people that there is actually very little misinformation online.

A.3.1 US

Figure A22: Supplementary Figure 22: Contextualized Media Coverage

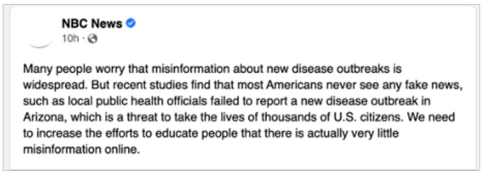

NBC News
10h ·

Many people worry that misinformation about new disease outbreaks is widespread. But recent studies find that most Americans never see any fake news, such as local public health officials failed to report a new disease outbreak in Arizona, which is a threat to take the lives of thousands of U.S. citizens. We need to increase the efforts to educate people that there is actually very little misinformation online.

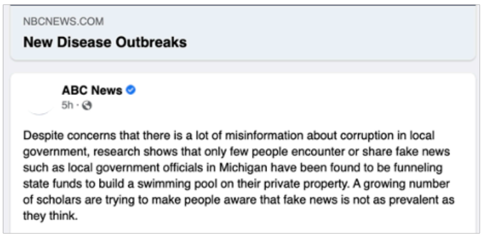

NBCNEWS.COM
New Disease Outbreaks

ABC News
5h ·

Despite concerns that there is a lot of misinformation about corruption in local government, research shows that only few people encounter or share fake news such as local government officials in Michigan have been found to be funneling state funds to build a swimming pool on their private property. A growing number of scholars are trying to make people aware that fake news is not as prevalent as they think.

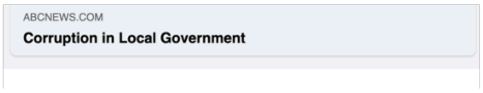

ABCNEWS.COM
Corruption in Local Government

Figure A23: Supplementary Figure 23: Decontextualized Media Coverage

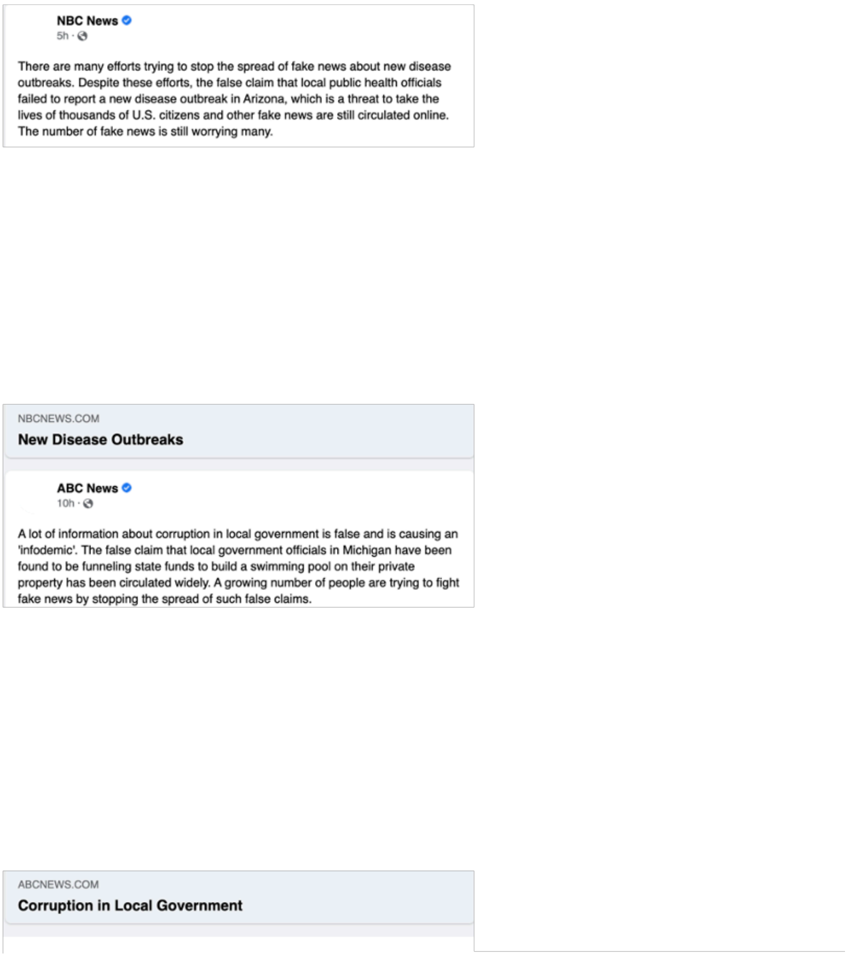

A.3.2 Poland

Figure A24: Supplementary Figure 24: Contextualized Media Coverage

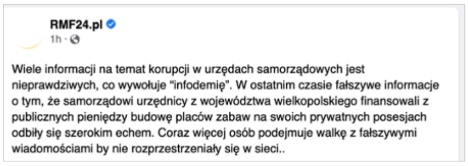

RMF24.pl
1h ·

Wiele informacji na temat korupcji w urzędach samorządowych jest nieprawdziwych, co wywołuje "infodemię". W ostatnim czasie fałszywe informacje o tym, że samorządowi urzędnicy z województwa wielkopolskiego finansowali z publicznych pieniędzy budowę placów zabaw na swoich prywatnych posesjach odbiły się szerokim echem. Coraz więcej osób podejmuje walkę z fałszywymi wiadomościami by nie rozprzestrzeniały się w sieci..

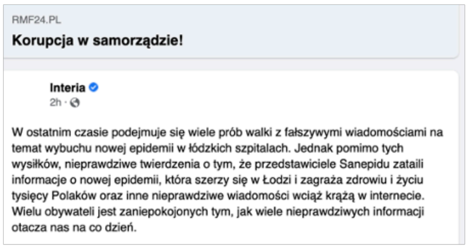

RMF24.PL
Korupcja w samorządzie!

Interia
2h ·

W ostatnim czasie podejmuje się wiele prób walki z fałszywymi wiadomościami na temat wybuchu nowej epidemii w łódzkich szpitalach. Jednak pomimo tych wysiłków, nieprawdziwe twierdzenia o tym, że przedstawiciele Sanepidu zataili informacje o nowej epidemii, która szerzy się w Łodzi i zagraża zdrowiu i życiu tysięcy Polaków oraz inne nieprawdziwe wiadomości wciąż krążą w internecie. Wielu obywateli jest zaniepokojonych tym, jak wiele nieprawdziwych informacji otacza nas na co dzień.

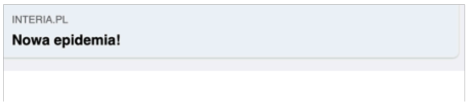

INTERIA.PL
Nowa epidemia!

Figure A25: Supplementary Figure 25: Decontextualized Media Coverage

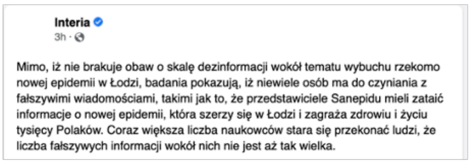

Interia
3h ·

Mimo, iż nie brakuje obaw o skalę dezinformacji wokół tematu wybuchu rzekomo nowej epidemii w Łodzi, badania pokazują, iż niewiele osób ma do czyniania z fałszywimi wiadomościami, takimi jak to, że przedstawiciele Sanepidu mieli zataić informacje o nowej epidemii, która szerzy się w Łodzi i zagraża zdrowiu i życiu tysięcy Polaków. Coraz większa liczba naukowców stara się przekonać ludzi, że liczba fałszywych informacji wokół nich nie jest aż tak wielka.

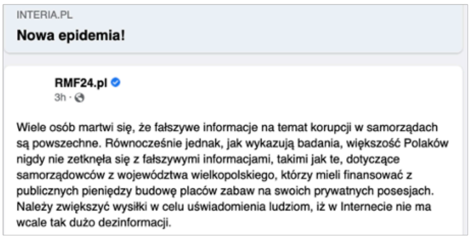

INTERIA.PL
Nowa epidemia!

RMF24.pl
3h ·

Wiele osób martwi się, że fałszywe informacje na temat korupcji w samorządach są powszechne. Równocześnie jednak, jak wykazują badania, większość Polaków nigdy nie zetknęła się z fałszywymi informacjami, takimi jak te, dotyczące samorządowców z województwa wielkopolskiego, którzy mieli finansować z publicznych pieniędzy budowę placów zabaw na swoich prywatnych posesjach. Należy zwiększyć wysiłki w celu uświadomienia ludziom, iż w Internecie nie ma wcale tak dużo dezinformacji.

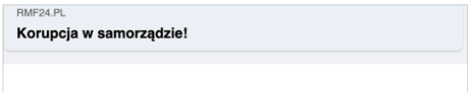

RMF24.PL
Korupcja w samorządzie!

A.3.3 Hong Kong

Figure A26: Supplementary Figure 26: Contextualized Media Coverage

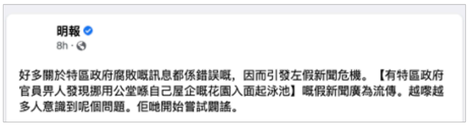

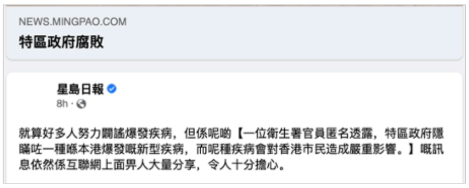

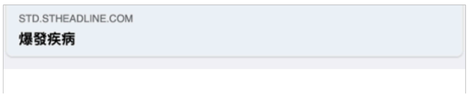

Figure A27: Supplementary Figure 27: Decontextualized Media Coverage

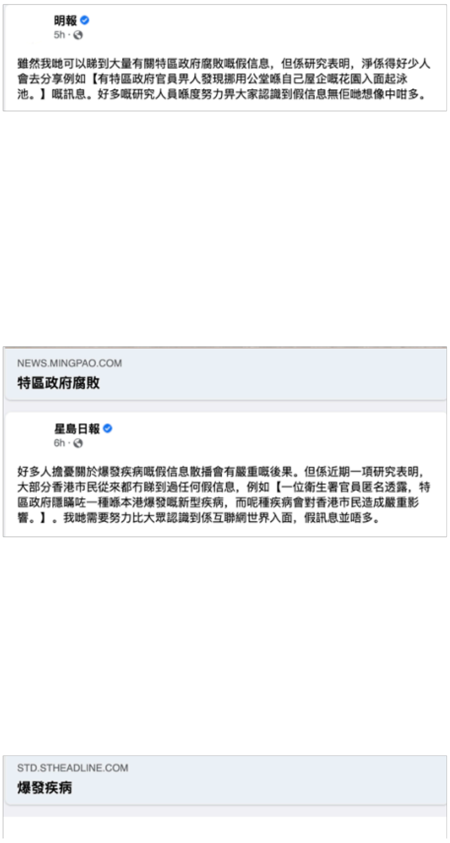

明報
5h ·

雖然我哋可以睇到大量有關特區政府腐敗嘅假信息，但係研究表明，淨係得好少人會去分享例如【有特區政府官員畀人發現挪用公堂喺自己屋企嘅花園入面起泳池。】嘅訊息。好多嘅研究人員喺度努力畀大家認識到假信息無佢哋想像中咁多。

NEWS.MINGPAO.COM
特區政府腐敗

星島日報
6h ·

好多人擔憂關於爆發疾病嘅假信息散播會有嚴重嘅後果。但係近期一項研究表明，大部分香港市民從來都冇睇到過任何假信息，例如【一位衛生署官員匿名透露，特區政府隱瞞咗一種喺本港爆發嘅新型疾病，而呢種疾病會對香港市民造成嚴重影響。】。我哋需要努力比大眾認識到係互聯網世界入面，假訊息並唔多。

STD.STHEADLINE.COM
爆發疾病

B Survey Measures

B.1 Control Variables

The following variables are measured in the pre-survey only (i.e. not in the post-survey, and not in the follow-up survey in the US):

- Gender ("How do you describe yourself?" Male Female Other)
- Age ("Which of the following categories includes your current age" with seven age categories)
- Race ("How do you describe yourself" with seven races)
- Education ("What is the highest level of school you have completed?" with seven levels)
- Ideology ("How would you rate yourself on this scale?" very liberal - very conservative; 5-point scale)
- Party identification ("Please select the option that best describes your political party affiliation" a strong Democrat - a strong Republican; 7-point scale)
- Political interest ("How interested are you in politics?" not at all - extremely; 5-point scale)
- Attention check ("To check whether you are reading the questions, click the second answer from the top" with 6 options)

Participants who fail the attention-check will be replaced by new participants by the polling companies and their data was not be analyzed.

B.2 Outcome Variables

The following variables were measured in the post-treatment survey in each country and in the follow-up survey in the US:

- **Misperceptions (2 false statements) & Skepticism (3 accurate statements):** Participants are asked how accurate they think each of the 5 statements are to the best of their knowledge (4-point scale)
- **Trust (7 issues):** Participants are asked to report how much they trust various institutions (7-point scale)

B.2.1 Statements US

- False Statement 1: Local government officials in Michigan have been found to be funneling state funds to build a swimming pool on their private property.
- False Statement 2: Local public health officials failed to report a new disease outbreak in Arizona, which threatens to take the lives of thousands of U.S. citizens.
- Factual Statement 1: In the US, roughly nine-in-ten adults get at least some news online (either via mobile or desktop)
- Factual Statement 2: Between February and June 2020, the share of young adults who are neither enrolled in school nor employed has more than doubled
- Factual Statement 3: Over 70% of the US population has a social media account

B.2.2 Statements Poland

- False Statement 1: Okazao si, e samorzdowi urzdnicy z województwa wielkopolskiego finansowali z publicznych pienidzy budow placów zabaw na swoich prywatnych posesjach.
- False Statement 2: Przedstawiciele ódzkiego Sanepidu zataili informacje o nowej epidemii, która szerzy si w odzi i zagraa zdrowiu i yciu tysicy obywateli Polski.
- Factual Statement 1: W listopadzie 2021 Urzd Komunikacji Elektronicznej przeprowadzi badanie klientów indywidualnych, z którego wynika, e a 96,9 proc. badanych korzysta z telefonu komórkowego, z czego a 78 proc. ze smartfona.
- Factual Statement 2: Jak podaje Gówny Urzd Statystyczny, najwiksze natenie bezrobocia ze wzgldu na wiek zaobserwowano wród osób modych w wieku od 15 do 24 lat. Dla tej grupy stopa bezrobocia wyniosa 14 proc.
- Factual Statement 3: 13 proc. modszych dzieci i a 15 proc. modziey jest uzalenionych od mediów spoecznociowych. Modzi chc ograniczy korzystanie z sieci, ale im si nie udaje, przez co zaniedbuj inne zajcia - wynika z ogólnopolskich bada jakoci ycia dzieci i modziey zleconych przez Rzecznika Praw Dziecka.

B.2.3 Statements Hong Kong

- False Statement 1: 喺自己屋企花園入面起泳池。
- False Statement 2: 名衛生署官員匿名透露，特區政府隱瞞咗一種喺本港爆發嘅新型疾病，而呢種疾病會對香港市民造成嚴重影響。

- Factual Statement 1: 事實陳述1: 有八成港人通過電視獲取新聞資訊。
- Factual Statement 2: 香港從舊年12月至今年2月嘅失業率為4.5%，較舊年11月至今年1月顯著上升0.6%。
- Factual Statement 3: 超過七成港人有社交媒體帳戶。

B.3 Consent and Debriefing Form

B.3.1 Consent Form

You are being asked to participate in a study conducted by researchers at the University of California Davis. You will be asked to look at two Facebook messages post by media organizations and Facebook users. The study will take about 8 minutes to complete. At no point will we ask you about your name or contact information. There are no risks to you or to your well-being. Your participation in this research study is voluntary. You can decide to stop the investigation at any time without giving us any reasons for your decision and without any negative consequences. If you decide not to participate in this study or if you withdraw from participating at any time, you will not be penalized.

Confidentiality

- Your name and any identifying information will not be collected.

Contact

- You can contact Magdalena Wojcieszak, Professor of Communication at the University of California, Davis [mwojcieszak@ucdavis.edu], or Brian Aitken [bcaitken@ucdavis.edu] if you have any questions or want more information about the study. Thank you very much for participating in this survey.

By clicking on the button below you confirm you

- have read the above information
- voluntarily agree to participate
- are at least 18 years of age or older

B.3.2 Debriefing Form

The session is almost complete but before you finish, it is important that you read the following information carefully.

The study was designed to test the effects of different misinformation correction strategies. As a participant, you were randomly assigned to one of the conditions with different instructions. Some of

you read mock social media posts and some of you did not read any information. If you remember these posts, please note that you read incorrect information that we, as researchers, wrote ourselves specifically for the purpose of this study in this survey. The claims made in the mock social media posts were purely made up to avoid any prior exposure that might influence the results of the study.

In order to make the study realistic, we did not tell you any of this at the beginning of the experiment. If you would like to discuss anything about this study or your participation in it, please contact the principal investigator, Emma Hoes [emma.hoes@uzh.ch].

B.4 Indices

For all aggregate measures in this study, all items that will result in an alpha-score lower than 0.7 will be omitted and analyzed separately (the results will be presented in supplementary materials).

- **Misperceptions:** We aggregated both the false items to create a single measure of misperceptions, where higher values indicate more belief in false statements.
- **Skepticism:** We aggregated the three true items to create a single measure of skepticism in verified facts, where higher values indicate less belief in accurate statements, hence higher levels of skepticism.
- **Trust:** We aggregated all seven trust items to create a single measure, with higher levels indicated more trust in several institutions.

B.5 Manipulation Check

To make sure any observed treatment effects are due to our manipulations, we presented participants with a statement depending on the treatment they were assigned to.

Those assigned to condition one and two (fact-checking) were presented with the following statement:

- **The posts I just saw mentioned the name of a concrete politician.** Participants have to indicate whether this statement is right or wrong. If those assigned to condition one answer 'right' and those assigned to condition two answer 'wrong', we conclude that manipulation has been successful.

Those assigned to condition three and four (media literacy) were presented with the following statement:

- **The posts I just saw presented tips for how to spot fake news.** Participants have to indicate whether this statement is right or wrong. If those assigned to condition three answer

'right' and those assigned to condition four answer 'wrong', we conclude that manipulation has been successful.

Those assigned to condition five and six (coverage of misinformation) were presented with the following statement:

- **The posts I just saw mentioned findings of academic research on the actual prevalence of misinformation.** Participants have to indicate whether this statement is right or wrong. If those assigned to condition five answer 'right' and those assigned to condition six answer 'wrong', we conclude that manipulation has been successful.

B.5.1 Demographic Comparisons between Compliers and Non-Compliers t-test Results

Figure B28: Supplementary Table 1: US Compliers and Non-Compliers t-test Results

Dependent Variable	*t*	*df*	*p*	*d*	95% CI
gender	-1.87	1,519.55	.062	-0.09	[-0.18, 0.00]
age	1.61	1,519.39	.107	0.07	[-0.02, 0.17]
race	-0.10	1,516.79	.923	-0.00	[-0.10, 0.09]
education	1.63	1,504.24	.104	0.08	[-0.02, 0.17]
ideology	0.85	1,499.76	.394	0.04	[-0.05, 0.13]

two-tailed t-test

Figure B29: Supplementary Table 2: PL Compliers and Non-Compliers t-test Results

Dependent Variable	*t*	*df*	*p*	*d*	95% CI
gender	-0.03	1,221.66	.980	-0.00	[-0.09, 0.09]
age	-0.02	1,204.36	.981	-0.00	[-0.09, 0.09]
municipal_size	-0.56	1,209.78	.573	-0.03	[-0.12, 0.07]
education	0.90	1,235.04	.369	0.04	[-0.05, 0.13]
ideology	0.80	1,234.27	.422	0.04	[-0.05, 0.13]

Note: two-tailed t-test

Figure B30: Supplementary Table 3: HK Compliers and Non-Compliers t-test Results

Dependent Variable	*t*	*df*	*p*	*d*	95% CI
age	-0.55	1,609.07	.581	-0.03	[-0.12, 0.07]
education	0.52	1,579.33	.601	0.02	[-0.07, 0.12]
residency	-0.55	1,466.97	.584	-0.03	[-0.12, 0.07]
birth_place	-1.04	1,473.80	.296	-0.05	[-0.14, 0.04]
party	0.06	1,572.92	.955	0.00	[-0.09, 0.09]

Note: two-tailed t-test

C Regression Tables

C.1 Main Results

C.1.1 Main Results - United States

Figure C31: Supplementary Table 4: Misperception by Treatment Regression in the US

Term	β	*SE*	*t*	*p*	95% CI
(Intercept)	3.02	0.13	22.62	< .001***	[2.76, 3.28]
age	-0.12	0.01	-10.14	< .001***	[-0.14, -0.10]
gender	-0.05	0.04	-1.53	.127	[-0.13, 0.02]
race	-0.02	0.02	-1.33	.185	[-0.06, 0.01]
education	-0.03	0.02	-1.63	.102	[-0.06, 0.01]
party ID	-0.01	0.01	-0.98	.329	[-0.03, 0.01]
Contextualized Media Coverage	-0.27	0.07	-4.03	< .001***	[-0.40, -0.14]
De-Contextualized Media Coverage	-0.14	0.07	-2.12	.034*	[-0.27, -0.01]
Accountability Fact Checking	-0.35	0.07	-5.39	< .001***	[-0.48, -0.22]
Correctability Fact Checking	-0.37	0.07	-5.51	< .001***	[-0.51, -0.24]
Bias News Media Literacy	-0.01	0.07	-0.09	.925	[-0.14, 0.13]
Fake News Media Literacy	-0.14	0.07	-2.07	.038*	[-0.27, -0.01]
political interest	0.05	0.02	3.45	.001***	[0.02, 0.09]

Note. * p < .05, ** p < .01, *** p < .001

Note: two-tailed regression test with no adjustments made for multiple comparisons

Figure C32: Supplementary Table 5: Skepticism by Treatment Regression in the US

Term	β	*SE*	*t*	*p*	95% CI
(Intercept)	2.03	0.10	20.53	< .001***	[1.83, 2.22]
age	0.10	0.01	11.54	< .001***	[0.08, 0.12]
gender	-0.06	0.03	-2.19	.029*	[-0.11, -0.01]
race	-0.01	0.01	-0.88	.379	[-0.04, 0.01]
education	0.02	0.01	1.87	.062	[-0.00, 0.04]
party ID	-0.00	0.01	-0.07	.943	[-0.01, 0.01]
Contextualized Media Coverage	0.13	0.05	2.65	.008**	[0.03, 0.23]
De-Contextualized Media Coverage	0.12	0.05	2.51	.012*	[0.03, 0.22]
Accountability Fact Checking	0.11	0.05	2.19	.029*	[0.01, 0.20]
Correctability Fact Checking	0.08	0.05	1.58	.115	[-0.02, 0.18]
Bias News Media Literacy	0.10	0.05	1.95	.051	[-0.00, 0.19]
Fake News Media Literacy	0.04	0.05	0.82	.412	[-0.06, 0.14]
political interest	-0.09	0.01	-7.29	< .001***	[-0.11, -0.06]

Note. * p < .05, ** p < .01, *** p < .001

Note: two-tailed regression test with no adjustments made for multiple comparisons

Figure C33: Supplementary Table 6: Trust by Treatment Regression in the US

Term	β	*SE*	*t*	*p*	95% CI
(Intercept)	4.61	0.19	24.02	< .001***	[4.23, 4.98]
age	-0.10	0.02	-6.14	< .001***	[-0.14, -0.07]
gender	-0.09	0.05	-1.82	.068	[-0.20, 0.01]
race	-0.00	0.03	-0.09	.930	[-0.05, 0.05]
education	0.12	0.02	5.17	< .001***	[0.07, 0.16]
party ID	-0.29	0.01	-22.27	< .001***	[-0.31, -0.26]
Contextualized Media Coverage	0.05	0.10	0.52	.606	[-0.14, 0.24]
De-Contextualized Media Coverage	-0.07	0.10	-0.72	.469	[-0.26, 0.12]
Accountability Fact Checking	0.17	0.09	1.83	.068	[-0.01, 0.36]
Correctability Fact Checking	-0.04	0.10	-0.39	.695	[-0.23, 0.15]
Bias News Media Literacy	-0.05	0.10	-0.56	.573	[-0.24, 0.14]
Fake News Media Literacy	0.03	0.10	0.30	.765	[-0.16, 0.22]
political interest	0.15	0.02	6.35	< .001***	[0.10, 0.19]

Note. * p < .05, ** p < .01, *** p < .001

Note: two-tailed regression test with no adjustments made for multiple comparisons

Figure C34: Supplementary Table 7: Trust in Journalists by Treatment Condition in the US

Term	β	*SE*	*t*	*p*	95% CI
(Intercept)	4.68	0.26	18.29	< .001***	[4.18, 5.18]
age	-0.07	0.02	-3.26	.001**	[-0.12, -0.03]
gender	-0.01	0.07	-0.17	.865	[-0.15, 0.12]
race	-0.06	0.04	-1.58	.113	[-0.13, 0.01]
education	0.08	0.03	2.58	.010**	[0.02, 0.14]
party ID	-0.33	0.02	-19.22	< .001***	[-0.36, -0.30]
Contextualized Media Coverage	-0.05	0.13	-0.38	.702	[-0.30, 0.20]
De-Contextualized Media Coverage	-0.16	0.13	-1.23	.221	[-0.41, 0.09]
Accountability Fact Checking	0.08	0.13	0.67	.506	[-0.16, 0.33]
Correctability Fact Checking	-0.15	0.13	-1.19	.235	[-0.41, 0.10]
Bias News Media Literacy	-0.09	0.13	-0.72	.474	[-0.35, 0.16]
Fake News Media Literacy	0.03	0.13	0.20	.838	[-0.23, 0.28]
political interest	0.20	0.03	6.50	< .001***	[0.14, 0.26]

Note. * p < .05, ** p < .01, *** p < .001

Note: two-tailed regression test with no adjustments made for multiple comparisons

Figure C35: Supplementary Table 8: Trust in Fact Checkers by Treatment Condition in the US

Term	β	*SE*	*t*	*p*	95% CI
(Intercept)	4.75	0.27	17.94	< .001***	[4.23, 5.27]
age	-0.10	0.02	-4.22	< .001***	[-0.14, -0.05]
gender	-0.04	0.07	-0.52	.605	[-0.18, 0.10]
race	0.07	0.04	1.81	.071	[-0.01, 0.14]
education	0.15	0.03	4.74	< .001***	[0.09, 0.21]
party ID	-0.35	0.02	-19.84	< .001***	[-0.39, -0.32]
Contextualized Media Coverage	0.13	0.13	0.96	.338	[-0.13, 0.39]
De-Contextualized Media Coverage	-0.17	0.13	-1.26	.209	[-0.43, 0.09]
Accountability Fact Checking	0.29	0.13	2.20	.028*	[0.03, 0.54]
Correctability Fact Checking	0.04	0.13	0.27	.790	[-0.23, 0.30]
Bias News Media Literacy	-0.04	0.13	-0.29	.772	[-0.30, 0.22]
Fake News Media Literacy	0.04	0.13	0.27	.786	[-0.23, 0.30]
political interest	0.13	0.03	4.08	< .001***	[0.07, 0.19]

Note. * p < .05, ** p < .01, *** p < .001

Note: two-tailed regression test with no adjustments made for multiple comparisons

Figure C36: Supplementary Table 9: Trust in US Government by Treatment Condition in the US

Term	β	*SE*	*t*	*p*	95% CI
(Intercept)	3.72	0.27	13.79	< .001***	[3.19, 4.25]
age	-0.02	0.02	-0.70	.487	[-0.06, 0.03]
gender	-0.22	0.07	-3.04	.002**	[-0.36, -0.08]
race	-0.01	0.04	-0.31	.759	[-0.08, 0.06]
education	0.19	0.03	5.96	< .001***	[0.13, 0.25]
party ID	-0.31	0.02	-16.93	< .001***	[-0.34, -0.27]
Contextualized Media Coverage	0.06	0.14	0.45	.651	[-0.21, 0.33]
De-Contextualized Media Coverage	-0.05	0.14	-0.33	.738	[-0.31, 0.22]
Accountability Fact Checking	0.21	0.13	1.63	.104	[-0.04, 0.47]
Correctability Fact Checking	-0.06	0.14	-0.46	.648	[-0.33, 0.21]
Bias News Media Literacy	-0.14	0.14	-1.06	.288	[-0.41, 0.12]
Fake News Media Literacy	0.11	0.14	0.81	.416	[-0.16, 0.38]
political interest	0.20	0.03	6.26	< .001***	[0.14, 0.26]

Note. * p < .05, ** p < .01, *** p < .001

Note: two-tailed regression test with no adjustments made for multiple comparisons

Figure C37: Supplementary Table 10: Trust in University Professors by Treatment Condition in the US

Term	β	*SE*	*t*	*p*	95% CI
(Intercept)	5.30	0.25	21.06	< .001***	[4.81, 5.80]
age	-0.19	0.02	-8.72	< .001***	[-0.24, -0.15]
gender	-0.09	0.07	-1.33	.183	[-0.22, 0.04]
race	0.02	0.03	0.64	.523	[-0.05, 0.09]
education	0.16	0.03	5.51	< .001***	[0.11, 0.22]
party ID	-0.29	0.02	-17.17	< .001***	[-0.32, -0.26]
Contextualized Media Coverage	0.18	0.13	1.38	.168	[-0.07, 0.42]
De-Contextualized Media Coverage	0.05	0.13	0.43	.669	[-0.19, 0.30]
Accountability Fact Checking	0.28	0.12	2.24	.026*	[0.03, 0.52]
Correctability Fact Checking	0.02	0.13	0.19	.848	[-0.23, 0.28]
Bias News Media Literacy	-0.08	0.13	-0.61	.539	[-0.33, 0.17]
Fake News Media Literacy	0.17	0.13	1.31	.190	[-0.08, 0.42]
political interest	0.06	0.03	1.85	.065	[-0.00, 0.11]

Note. * p < .05, ** p < .01, *** p < .001

Note: two-tailed regression test with no adjustments made for multiple comparisons

Figure C38: Supplementary Table 11: Trust in Social Media by Treatment Condition in the US

Term	β	*SE*	*t*	*p*	95% CI
(Intercept)	4.55	0.25	18.01	< .001***	[4.06, 5.05]
age	-0.22	0.02	-9.76	< .001***	[-0.26, -0.17]
gender	-0.08	0.07	-1.12	.263	[-0.21, 0.06]
race	-0.17	0.03	-4.86	< .001***	[-0.24, -0.10]
education	0.01	0.03	0.50	.620	[-0.04, 0.07]
party ID	-0.12	0.02	-7.15	< .001***	[-0.16, -0.09]
Contextualized Media Coverage	-0.04	0.13	-0.29	.776	[-0.29, 0.21]
De-Contextualized Media Coverage	-0.02	0.13	-0.14	.889	[-0.27, 0.23]
Accountability Fact Checking	0.08	0.12	0.67	.504	[-0.16, 0.33]
Correctability Fact Checking	0.04	0.13	0.27	.785	[-0.22, 0.29]
Bias News Media Literacy	-0.01	0.13	-0.09	.930	[-0.26, 0.24]
Fake News Media Literacy	-0.07	0.13	-0.53	.598	[-0.32, 0.18]
political interest	0.14	0.03	4.79	< .001***	[0.09, 0.20]

Note. * p < .05, ** p < .01, *** p < .001

Note: two-tailed regression test with no adjustments made for multiple comparisons

Figure C39: Supplementary Table 12: Trust in Traditional Media by Treatment Condition in the US

Term	β	*SE*	*t*	*p*	95% CI
(Intercept)	3.87	0.26	14.75	< .001***	[3.36, 4.39]
age	-0.01	0.02	-0.46	.649	[-0.06, 0.03]
gender	-0.04	0.07	-0.63	.526	[-0.18, 0.09]
race	0.00	0.04	0.10	.924	[-0.07, 0.07]
education	0.10	0.03	3.25	.001**	[0.04, 0.16]
party ID	-0.32	0.02	-18.26	< .001***	[-0.36, -0.29]
Contextualized Media Coverage	0.13	0.13	1.00	.319	[-0.13, 0.39]
De-Contextualized Media Coverage	-0.01	0.13	-0.09	.929	[-0.27, 0.25]
Accountability Fact Checking	0.23	0.13	1.78	.075	[-0.02, 0.48]
Correctability Fact Checking	0.00	0.13	0.03	.974	[-0.26, 0.27]
Bias News Media Literacy	0.03	0.13	0.23	.818	[-0.23, 0.29]
Fake News Media Literacy	0.01	0.13	0.04	.966	[-0.26, 0.27]
political interest	0.18	0.03	5.73	< .001***	[0.12, 0.24]

Note. * p < .05, ** p < .01, *** p < .001

Note: two-tailed regression test with no adjustments made for multiple comparisons

Figure C40: Supplementary Table 13: Trust in Scientists by Treatment Condition in the US

Term	β	*SE*	*t*	*p*	95% CI
(Intercept)	5.37	0.24	22.42	< .001***	[4.90, 5.84]
age	-0.12	0.02	-5.52	< .001***	[-0.16, -0.07]
gender	-0.18	0.06	-2.77	.006**	[-0.30, -0.05]
race	0.13	0.03	3.90	< .001***	[0.06, 0.19]
education	0.12	0.03	4.37	< .001***	[0.07, 0.18]
party ID	-0.28	0.02	-17.69	< .001***	[-0.32, -0.25]
Contextualized Media Coverage	-0.06	0.12	-0.51	.611	[-0.30, 0.18]
De-Contextualized Media Coverage	-0.14	0.12	-1.18	.236	[-0.38, 0.09]
Accountability Fact Checking	0.03	0.12	0.26	.798	[-0.20, 0.26]
Correctability Fact Checking	-0.15	0.12	-1.23	.218	[-0.39, 0.09]
Bias News Media Literacy	-0.05	0.12	-0.40	.693	[-0.28, 0.19]
Fake News Media Literacy	-0.08	0.12	-0.63	.528	[-0.31, 0.16]
political interest	0.11	0.03	3.80	< .001***	[0.05, 0.16]

Note. * p < .05, ** p < .01, *** p < .001

Note: two-tailed regression test with no adjustments made for multiple comparisons

Figure C41: Supplementary Table 14: Misperception Regression comparing Accountability and Correctability Strategies in the US

Term	β	*SE*	*t*	*p*	95% CI
(Intercept)	2.66	0.20	13.08	< .001***	[2.26, 3.06]
age	-0.14	0.02	-7.61	< .001***	[-0.18, -0.11]
gender	-0.06	0.06	-1.03	.301	[-0.17, 0.05]
race	-0.01	0.03	-0.50	.618	[-0.07, 0.04]
education	-0.02	0.03	-0.63	.532	[-0.06, 0.03]
party ID	-0.01	0.01	-0.56	.576	[-0.04, 0.02]
Control	0.35	0.07	5.22	< .001***	[0.22, 0.49]
Correctability Fact Checking	-0.02	0.07	-0.34	.731	[-0.16, 0.11]
political interest	0.06	0.02	2.31	.021*	[0.01, 0.10]

Note. * p < .05, ** p < .01, *** p < .001

Note: two-tailed regression test with no adjustments made for multiple comparisons

Figure C42: Supplementary Table 15: Skepticism Regression comparing Accountability and Correctability Strategies in the US

Term	β	*SE*	*t*	*p*	95% CI
(Intercept)	2.27	0.15	15.64	< .001***	[1.99, 2.56]
age	0.10	0.01	7.70	< .001***	[0.08, 0.13]
gender	-0.06	0.04	-1.56	.119	[-0.14, 0.02]
race	-0.03	0.02	-1.34	.181	[-0.07, 0.01]
education	0.02	0.02	1.24	.217	[-0.01, 0.06]
party ID	-0.00	0.01	-0.20	.845	[-0.02, 0.02]
Control	-0.11	0.05	-2.23	.026*	[-0.20, -0.01]
Correctability Fact Checking	-0.03	0.05	-0.56	.575	[-0.12, 0.07]
political interest	-0.10	0.02	-5.84	< .001***	[-0.14, -0.07]

Note. * p < .05, ** p < .01, *** p < .001

Note: two-tailed regression test with no adjustments made for multiple comparisons

Figure C43: Supplementary Table 16: Trust Regression comparing Accountability and Correctability Strategies in the US

Term	β	*SE*	*t*	*p*	95% CI
(Intercept)	4.83	0.29	16.75	< .001***	[4.26, 5.39]
age	-0.10	0.03	-3.60	< .001***	[-0.15, -0.04]
gender	-0.19	0.08	-2.39	.017*	[-0.35, -0.03]
race	0.04	0.04	0.86	.391	[-0.05, 0.12]
education	0.09	0.04	2.67	.008**	[0.03, 0.16]
party ID	-0.29	0.02	-14.32	< .001***	[-0.33, -0.25]
Control	-0.17	0.10	-1.77	.077	[-0.36, 0.02]
Correctability Fact Checking	-0.22	0.10	-2.28	.023*	[-0.41, -0.03]
political interest	0.13	0.03	3.72	< .001***	[0.06, 0.20]

Note. * p < .05, ** p < .01, *** p < .001

Note: two-tailed regression test with no adjustments made for multiple comparisons

Figure C44: Supplementary Table 17: Skepticism Regression comparing Bias Focus and Fake News Focus Strategies in the US

Term	β	*SE*	*t*	*p*	95% CI
(Intercept)	2.31	0.14	16.27	< .001***	[2.03, 2.58]
age	0.09	0.01	6.88	< .001***	[0.06, 0.12]
gender	-0.06	0.04	-1.56	.119	[-0.14, 0.02]
race	-0.03	0.02	-1.51	.130	[-0.07, 0.01]
education	0.01	0.02	0.63	.527	[-0.02, 0.05]
party ID	-0.01	0.01	-0.87	.385	[-0.03, 0.01]
Control	-0.09	0.05	-1.93	.054	[-0.19, 0.00]
Fake News Media Literacy	-0.05	0.05	-0.95	.341	[-0.14, 0.05]
political interest	-0.08	0.02	-4.51	< .001***	[-0.11, -0.05]

Note. * p < .05, ** p < .01, *** p < .001

Note: two-tailed regression test with no adjustments made for multiple comparisons

Figure C45: Supplementary Table 18: Trust Regression comparing Bias Focus and Fake News Focus Strategies in the US

Term	β	*SE*	*t*	*p*	95% CI
(Intercept)	4.62	0.29	16.16	< .001***	[4.06, 5.18]
age	-0.12	0.03	-4.71	< .001***	[-0.18, -0.07]
gender	-0.01	0.08	-0.18	.861	[-0.17, 0.14]
race	-0.05	0.04	-1.31	.190	[-0.13, 0.03]
education	0.12	0.04	3.33	.001***	[0.05, 0.19]
party ID	-0.28	0.02	-13.88	< .001***	[-0.32, -0.24]
Control	0.06	0.10	0.59	.558	[-0.13, 0.25]
Fake News Media Literacy	0.09	0.10	0.96	.338	[-0.10, 0.28]
political interest	0.18	0.04	5.04	< .001***	[0.11, 0.25]

Note. * p < .05, ** p < .01, *** p < .001

Note: two-tailed regression test with no adjustments made for multiple comparisons

Figure C46: Supplementary Table 19: Misperception Regression comparing Contextualized and De-Contextualized Strategies in the US

Term	β	*SE*	*t*	*p*	95% CI
(Intercept)	2.80	0.19	15.01	< .001***	[2.44, 3.17]
age	-0.11	0.02	-6.27	< .001***	[-0.15, -0.08]
gender	-0.04	0.05	-0.66	.508	[-0.14, 0.07]
race	-0.03	0.03	-1.30	.193	[-0.09, 0.02]
education	-0.02	0.02	-0.78	.433	[-0.07, 0.03]
party ID	-0.01	0.01	-1.00	.316	[-0.04, 0.01]
Control	0.27	0.07	4.05	< .001***	[0.14, 0.40]
De-Contextualized Media Coverage	0.13	0.07	1.95	.051	[-0.00, 0.25]
political interest	0.04	0.02	1.58	.116	[-0.01, 0.08]

Note. * p < .05, ** p < .01, *** p < .001

Note: two-tailed regression test with no adjustments made for multiple comparisons

Figure C47: Supplementary Table 20: Skepticism Regression comparing Contextualized and De-Contextualized Strategies in the US

Term	β	*SE*	*t*	*p*	95% CI
(Intercept)	1.73	0.14	12.04	< .001***	[1.45, 2.01]
age	0.10	0.01	7.73	< .001***	[0.08, 0.13]
gender	0.01	0.04	0.29	.774	[-0.07, 0.09]
race	0.02	0.02	0.76	.450	[-0.02, 0.06]
education	0.01	0.02	0.42	.678	[-0.03, 0.04]
party ID	0.00	0.01	0.06	.954	[-0.02, 0.02]
Control	0.13	0.05	2.62	.009**	[0.03, 0.23]
De-Contextualized Media Coverage	0.13	0.05	2.50	.013*	[0.03, 0.22]
political interest	-0.07	0.02	-3.64	< .001***	[-0.10, -0.03]

Note. * p < .05, ** p < .01, *** p < .001

Figure C48: Supplementary Table 21: Trust Regression comparing Contextualized and De-Contextualized Strategies in the US

Term	β	*SE*	*t*	*p*	95% CI
(Intercept)	4.65	0.28	16.86	< .001***	[4.11, 5.19]
age	-0.09	0.03	-3.52	< .001***	[-0.14, -0.04]
gender	-0.04	0.08	-0.54	.592	[-0.20, 0.11]
race	0.01	0.04	0.27	.791	[-0.07, 0.09]
education	0.11	0.04	3.06	.002**	[0.04, 0.18]
party ID	-0.30	0.02	-14.48	< .001***	[-0.34, -0.26]
Control	-0.06	0.10	-0.56	.573	[-0.25, 0.14]
De-Contextualized Media Coverage	-0.13	0.10	-1.33	.183	[-0.32, 0.06]
political interest	0.11	0.04	2.98	.003**	[0.04, 0.18]

Note. * p < .05, ** p < .01, *** p < .001

Note: two-tailed regression test with no adjustments made for multiple comparisons

C.1.2 Main Results - Poland

Figure C49: Supplementary Table 22: Misperception by Treatment Regression in Poland

Term	β	*SE*	*t*	*p*	95% CI
(Intercept)	2.33	0.10	24.14	< .001***	[2.14, 2.52]
age	-0.06	0.01	-5.95	< .001***	[-0.08, -0.04]
gender	0.05	0.03	1.57	.117	[-0.01, 0.11]
race	0.02	0.01	2.23	.026*	[0.00, 0.04]
education	-0.02	0.01	-2.12	.034*	[-0.04, -0.00]
support oppose	0.01	0.00	2.03	.042*	[0.00, 0.02]
Contextualized Media Coverage	-0.07	0.06	-1.28	.202	[-0.18, 0.04]
De-Contextualized Media Coverage	0.01	0.06	0.19	.852	[-0.10, 0.12]
Accountability Fact Checking	-0.21	0.06	-3.77	< .001***	[-0.32, -0.10]
Correctability Fact Checking	-0.29	0.05	-5.51	< .001***	[-0.39, -0.19]
Bias News Media Literacy	-0.08	0.06	-1.42	.156	[-0.19, 0.03]
Fake News Media Literacy	0.01	0.05	0.14	.886	[-0.10, 0.11]
political interest	-0.04	0.02	-2.17	.030*	[-0.07, -0.00]

Note. * p < .05, ** p < .01, *** p < .001

Note: two-tailed regression test with no adjustments made for multiple comparisons

Figure C50: Supplementary Table 23: Skepticism by Treatment Regression in Poland

Term	β	*SE*	*t*	*p*	95% CI
(Intercept)	2.25	0.08	28.86	< .001***	[2.10, 2.41]
age	0.01	0.01	1.75	.080	[-0.00, 0.03]
gender	-0.03	0.02	-1.06	.288	[-0.07, 0.02]
race	0.01	0.01	1.71	.087	[-0.00, 0.03]
education	-0.01	0.01	-1.00	.316	[-0.02, 0.01]
support oppose	-0.00	0.00	-0.08	.935	[-0.01, 0.01]
Contextualized Media Coverage	0.14	0.05	3.06	.002**	[0.05, 0.23]
De-Contextualized Media Coverage	0.13	0.05	2.80	.005**	[0.04, 0.22]
Accountability Fact Checking	0.08	0.05	1.74	.083	[-0.01, 0.17]
Correctability Fact Checking	0.16	0.04	3.72	< .001***	[0.08, 0.24]
Bias News Media Literacy	0.16	0.04	3.59	< .001***	[0.07, 0.25]
Fake News Media Literacy	0.03	0.04	0.62	.534	[-0.06, 0.11]
political interest	-0.04	0.01	-3.29	.001**	[-0.07, -0.02]

Note. * p < .05, ** p < .01, *** p < .001

Note: two-tailed regression test with no adjustments made for multiple comparisons

Figure C51: Supplementary Table 24: Trust by Treatment Regression in Poland

Term	β	*SE*	*t*	*p*	95% CI
(Intercept)	2.83	0.12	23.09	< .001***	[2.59, 3.07]
age	-0.02	0.01	-1.57	.117	[-0.04, 0.00]
gender	0.10	0.04	2.55	.011*	[0.02, 0.17]
race	0.01	0.01	0.64	.522	[-0.02, 0.03]
education	-0.01	0.01	-0.42	.674	[-0.03, 0.02]
support oppose	0.07	0.01	10.61	< .001***	[0.05, 0.08]
Contextualized Media Coverage	-0.07	0.07	-1.02	.309	[-0.21, 0.07]
De-Contextualized Media Coverage	-0.05	0.07	-0.71	.480	[-0.19, 0.09]
Accountability Fact Checking	-0.04	0.07	-0.58	.559	[-0.18, 0.10]
Correctability Fact Checking	0.11	0.07	1.59	.111	[-0.02, 0.24]
Bias News Media Literacy	-0.11	0.07	-1.55	.122	[-0.24, 0.03]
Fake News Media Literacy	-0.01	0.07	-0.17	.863	[-0.15, 0.12]
political interest	0.19	0.02	8.82	< .001***	[0.15, 0.23]

Note. * p < .05, ** p < .01, *** p < .001

Note: two-tailed regression test with no adjustments made for multiple comparisons

Figure C52: Supplementary Table 25: Trust in Journalists by Treatment Condition in Poland

Term	β	*SE*	*t*	*p*	95% CI
(Intercept)	2.68	0.18	14.48	< .001***	[2.32, 3.04]
age	0.08	0.02	4.45	< .001***	[0.05, 0.12]
gender	0.15	0.06	2.59	.010**	[0.04, 0.26]
race	-0.01	0.02	-0.29	.775	[-0.04, 0.03]
education	-0.06	0.02	-3.03	.002**	[-0.09, -0.02]
support oppose	-0.01	0.01	-1.05	.293	[-0.03, 0.01]
Contextualized Media Coverage	-0.05	0.11	-0.50	.618	[-0.26, 0.16]
De-Contextualized Media Coverage	-0.05	0.11	-0.48	.632	[-0.26, 0.16]
Accountability Fact Checking	-0.03	0.11	-0.30	.764	[-0.24, 0.18]
Correctability Fact Checking	0.12	0.10	1.22	.224	[-0.08, 0.32]
Bias News Media Literacy	-0.16	0.11	-1.50	.134	[-0.36, 0.05]
Fake News Media Literacy	0.00	0.10	0.03	.977	[-0.20, 0.21]
political interest	0.21	0.03	6.62	< .001***	[0.15, 0.27]

Note. * p < .05, ** p < .01, *** p < .001

Note: two-tailed regression test with no adjustments made for multiple comparisons

Figure C53: Supplementary Table 26: Trust in Fact Checkers by Treatment Condition in Poland

Term	β	*SE*	*t*	*p*	95% CI
(Intercept)	3.68	0.21	17.73	< .001***	[3.27, 4.09]
age	-0.17	0.02	-8.13	< .001***	[-0.21, -0.13]
gender	0.23	0.06	3.61	< .001***	[0.11, 0.36]
race	0.02	0.02	0.84	.401	[-0.03, 0.06]
education	0.00	0.02	0.01	.991	[-0.04, 0.04]
support oppose	-0.02	0.01	-2.27	.023*	[-0.04, -0.00]
Contextualized Media Coverage	-0.25	0.12	-2.09	.036*	[-0.49, -0.02]
De-Contextualized Media Coverage	-0.27	0.12	-2.22	.027*	[-0.51, -0.03]
Accountability Fact Checking	-0.16	0.12	-1.30	.193	[-0.39, 0.08]
Correctability Fact Checking	0.04	0.11	0.39	.694	[-0.18, 0.27]
Bias News Media Literacy	-0.10	0.12	-0.84	.400	[-0.33, 0.13]
Fake News Media Literacy	0.02	0.12	0.16	.874	[-0.21, 0.25]
political interest	0.22	0.04	6.03	< .001***	[0.15, 0.29]

Note. * p < .05, ** p < .01, *** p < .001

Note: two-tailed regression test with no adjustments made for multiple comparisons

Figure C54: Supplementary Table 27: Trust in PL Government by Treatment Condition in Poland

Term	β	*SE*	*t*	*p*	95% CI
(Intercept)	0.83	0.15	5.57	< .001***	[0.53, 1.12]
age	-0.02	0.01	-1.17	.243	[-0.05, 0.01]
gender	-0.10	0.05	-2.13	.034*	[-0.19, -0.01]
race	0.01	0.02	0.43	.668	[-0.02, 0.04]
education	-0.01	0.02	-0.62	.534	[-0.04, 0.02]
support oppose	0.41	0.01	54.85	< .001***	[0.39, 0.42]
Contextualized Media Coverage	0.01	0.09	0.12	.908	[-0.16, 0.18]
De-Contextualized Media Coverage	0.10	0.09	1.17	.241	[-0.07, 0.27]
Accountability Fact Checking	-0.02	0.09	-0.18	.855	[-0.18, 0.15]
Correctability Fact Checking	0.18	0.08	2.20	.028*	[0.02, 0.34]
Bias News Media Literacy	0.06	0.08	0.69	.493	[-0.11, 0.22]
Fake News Media Literacy	-0.01	0.08	-0.14	.890	[-0.17, 0.15]
political interest	0.14	0.03	5.54	< .001***	[0.09, 0.19]

Note. * p < .05, ** p < .01, *** p < .001

Note: two-tailed regression test with no adjustments made for multiple comparisons

Figure C55: Supplementary Table 28: Trust in University Professors by Treatment Condition in Poland

Term	β	*SE*	*t*	*p*	95% CI
(Intercept)	3.48	0.18	19.35	< .001***	[3.12, 3.83]
age	-0.03	0.02	-1.70	.089	[-0.07, 0.00]
gender	0.09	0.06	1.55	.122	[-0.02, 0.20]
race	0.02	0.02	1.16	.247	[-0.02, 0.06]
education	0.10	0.02	5.48	< .001***	[0.06, 0.14]
support oppose	-0.01	0.01	-0.87	.387	[-0.03, 0.01]
Contextualized Media Coverage	-0.02	0.10	-0.18	.860	[-0.22, 0.19]
De-Contextualized Media Coverage	-0.02	0.11	-0.15	.880	[-0.22, 0.19]
Accountability Fact Checking	0.02	0.10	0.17	.861	[-0.19, 0.22]
Correctability Fact Checking	0.04	0.10	0.44	.661	[-0.15, 0.24]
Bias News Media Literacy	-0.18	0.10	-1.79	.073	[-0.38, 0.02]
Fake News Media Literacy	0.00	0.10	0.04	.966	[-0.19, 0.20]
political interest	0.18	0.03	5.90	< .001***	[0.12, 0.24]

Note. * p < .05, ** p < .01, *** p < .001

Note: two-tailed regression test with no adjustments made for multiple comparisons

Figure C56: Supplementary Table 29: Trust in Social Media by Treatment Condition in Poland

Term	β	*SE*	*t*	*p*	95% CI
(Intercept)	2.90	0.19	15.66	< .001***	[2.53, 3.26]
age	-0.02	0.02	-0.86	.392	[-0.05, 0.02]
gender	0.25	0.06	4.40	< .001***	[0.14, 0.37]
race	0.00	0.02	0.05	.958	[-0.04, 0.04]
education	-0.12	0.02	-6.48	< .001***	[-0.16, -0.09]
support oppose	0.02	0.01	2.24	.026*	[0.00, 0.04]
Contextualized Media Coverage	-0.14	0.11	-1.30	.193	[-0.35, 0.07]
De-Contextualized Media Coverage	-0.06	0.11	-0.55	.580	[-0.27, 0.15]
Accountability Fact Checking	0.03	0.11	0.27	.790	[-0.18, 0.24]
Correctability Fact Checking	0.16	0.10	1.57	.117	[-0.04, 0.36]
Bias News Media Literacy	-0.16	0.11	-1.55	.121	[-0.37, 0.04]
Fake News Media Literacy	-0.13	0.10	-1.23	.218	[-0.33, 0.08]
political interest	0.14	0.03	4.26	< .001***	[0.07, 0.20]

Note. * p < .05, ** p < .01, *** p < .001

Note: two-tailed regression test with no adjustments made for multiple comparisons

Figure C57: Supplementary Table 30: Trust in Traditional Media by Treatment Condition in Poland

Term	β	*SE*	*t*	*p*	95% CI
(Intercept)	2.63	0.20	13.37	< .001***	[2.24, 3.01]
age	0.04	0.02	2.04	.041*	[0.00, 0.08]
gender	-0.07	0.06	-1.20	.230	[-0.19, 0.05]
race	-0.02	0.02	-0.86	.392	[-0.06, 0.02]
education	-0.05	0.02	-2.73	.006**	[-0.09, -0.02]
support oppose	0.11	0.01	11.40	< .001***	[0.09, 0.13]
Contextualized Media Coverage	-0.10	0.11	-0.86	.389	[-0.32, 0.13]
De-Contextualized Media Coverage	0.00	0.12	0.01	.996	[-0.23, 0.23]
Accountability Fact Checking	-0.03	0.11	-0.30	.765	[-0.26, 0.19]
Correctability Fact Checking	0.08	0.11	0.75	.456	[-0.13, 0.29]
Bias News Media Literacy	-0.05	0.11	-0.44	.660	[-0.27, 0.17]
Fake News Media Literacy	-0.01	0.11	-0.12	.904	[-0.23, 0.20]
political interest	0.18	0.03	5.26	< .001***	[0.11, 0.25]

Note. * p < .05, ** p < .01, *** p < .001

Note: two-tailed regression test with no adjustments made for multiple comparisons

Figure C58: Supplementary Table 31: Trust in Scientists by Treatment Condition in Poland

Term	β	*SE*	*t*	*p*	95% CI
(Intercept)	3.60	0.18	19.86	< .001***	[3.25, 3.96]
age	-0.02	0.02	-1.35	.178	[-0.06, 0.01]
gender	0.13	0.06	2.30	.021*	[0.02, 0.24]
race	0.03	0.02	1.73	.084	[-0.00, 0.07]
education	0.11	0.02	5.75	< .001***	[0.07, 0.14]
support oppose	-0.04	0.01	-4.79	< .001***	[-0.06, -0.03]
Contextualized Media Coverage	0.05	0.10	0.44	.663	[-0.16, 0.25]
De-Contextualized Media Coverage	-0.06	0.11	-0.56	.577	[-0.27, 0.15]
Accountability Fact Checking	-0.10	0.10	-0.94	.348	[-0.30, 0.11]
Correctability Fact Checking	0.12	0.10	1.20	.230	[-0.08, 0.31]
Bias News Media Literacy	-0.16	0.10	-1.54	.124	[-0.36, 0.04]
Fake News Media Literacy	0.04	0.10	0.43	.667	[-0.16, 0.24]
political interest	0.24	0.03	7.59	< .001***	[0.18, 0.30]

Note. * p < .05, ** p < .01, *** p < .001

Note: two-tailed regression test with no adjustments made for multiple comparisons

Figure C59: Supplementary Table 32: Misperception Regression comparing Accountability and Correctability Strategies in Poland

Term	β	*SE*	*t*	*p*	95% CI
(Intercept)	2.25	0.14	15.96	< .001***	[1.97, 2.52]
age	-0.07	0.01	-4.49	< .001***	[-0.09, -0.04]
gender	0.05	0.05	1.18	.239	[-0.04, 0.14]
race	0.00	0.02	0.30	.761	[-0.03, 0.04]
education	-0.01	0.01	-0.88	.381	[-0.04, 0.02]
support oppose	0.00	0.01	0.41	.682	[-0.01, 0.02]
Control	0.21	0.06	3.75	< .001***	[0.10, 0.32]
Correctability Fact Checking	-0.08	0.05	-1.56	.119	[-0.19, 0.02]
political interest	-0.06	0.03	-2.33	.020*	[-0.11, -0.01]

Note. * p < .05, ** p < .01, *** p < .001

Note: two-tailed regression test with no adjustments made for multiple comparisons

Figure C60: Supplementary Table 33: Skepticism Regression comparing Accountability and Correctability Strategies in Poland

Term	β	*SE*	*t*	*p*	95% CI
(Intercept)	2.31	0.11	20.61	< .001***	[2.09, 2.53]
age	0.02	0.01	1.46	.144	[-0.01, 0.04]
gender	-0.06	0.04	-1.52	.130	[-0.13, 0.02]
race	0.02	0.01	1.70	.089	[-0.00, 0.05]
education	0.00	0.01	0.16	.873	[-0.02, 0.03]
support oppose	0.00	0.01	0.17	.863	[-0.01, 0.01]
Control	-0.08	0.04	-1.79	.073	[-0.17, 0.01]
Correctability Fact Checking	0.08	0.04	1.84	.066	[-0.01, 0.16]
political interest	-0.05	0.02	-2.37	.018*	[-0.09, -0.01]

Note. * p < .05, ** p < .01, *** p < .001

Note: two-tailed regression test with no adjustments made for multiple comparisons

Figure C61: Supplementary Table 34: Trust Regression comparing Accountability and Correctability Strategies in Poland

Term	β	*SE*	*t*	*p*	95% CI
(Intercept)	2.66	0.17	15.69	< .001***	[2.32, 2.99]
age	-0.01	0.02	-0.38	.704	[-0.04, 0.03]
gender	0.13	0.06	2.33	.020*	[0.02, 0.24]
race	0.01	0.02	0.76	.450	[-0.02, 0.05]
education	-0.01	0.02	-0.67	.505	[-0.05, 0.02]
support oppose	0.07	0.01	7.71	< .001***	[0.05, 0.09]
Control	0.04	0.07	0.60	.547	[-0.09, 0.17]
Correctability Fact Checking	0.15	0.06	2.34	.019*	[0.02, 0.28]
political interest	0.20	0.03	6.58	< .001***	[0.14, 0.26]

Note. * p < .05, ** p < .01, *** p < .001

Note: two-tailed regression test with no adjustments made for multiple comparisons

Figure C62: Supplementary Table 35: Skepticism Regression comparing Bias Focus and Fake News Focus Strategies in Poland

Term	β	*SE*	*t*	*p*	95% CI
(Intercept)	2.33	0.11	20.94	< .001***	[2.11, 2.55]
age	0.01	0.01	1.17	.242	[-0.01, 0.04]
gender	-0.03	0.04	-0.91	.363	[-0.11, 0.04]
race	0.00	0.01	0.19	.847	[-0.02, 0.03]
education	0.00	0.01	0.31	.758	[-0.02, 0.03]
support oppose	0.00	0.01	0.01	.993	[-0.01, 0.01]
Control	-0.16	0.04	-3.65	< .001***	[-0.25, -0.08]
Fake News Media Literacy	-0.13	0.04	-3.01	.003**	[-0.22, -0.05]
political interest	-0.02	0.02	-1.01	.313	[-0.06, 0.02]

Note. * p < .05, ** p < .01, *** p < .001

Note: two-tailed regression test with no adjustments made for multiple comparisons

Figure C63: Supplementary Table 36: Trust Regression comparing Bias Focus and Fake News Focus Strategies in Poland

Term	β	*SE*	*t*	*p*	95% CI
(Intercept)	2.82	0.18	15.93	< .001***	[2.47, 3.16]
age	-0.03	0.02	-1.51	.132	[-0.07, 0.01]
gender	0.04	0.06	0.62	.537	[-0.08, 0.15]
race	0.00	0.02	0.20	.839	[-0.04, 0.04]
education	0.02	0.02	0.85	.398	[-0.02, 0.05]
support oppose	0.07	0.01	7.48	< .001***	[0.05, 0.09]
Control	0.11	0.07	1.57	.116	[-0.03, 0.25]
Fake News Media Literacy	0.09	0.07	1.36	.174	[-0.04, 0.23]
political interest	0.17	0.03	5.03	< .001***	[0.10, 0.23]

Note. * p < .05, ** p < .01, *** p < .001

Note: two-tailed regression test with no adjustments made for multiple comparisons

Figure C64: Supplementary Table 37: Misperception Regression comparing Contextualized and De-Contextualized Strategies in Poland

Term	β	*SE*	*t*	*p*	95% CI
(Intercept)	2.17	0.14	15.14	< .001***	[1.89, 2.46]
age	-0.06	0.02	-3.81	< .001***	[-0.09, -0.03]
gender	0.10	0.05	2.08	.038*	[0.01, 0.20]
race	0.02	0.02	1.00	.318	[-0.02, 0.05]
education	-0.03	0.02	-1.98	.048*	[-0.06, -0.00]
support oppose	0.01	0.01	0.79	.427	[-0.01, 0.02]
Control	0.07	0.06	1.27	.203	[-0.04, 0.18]
Correctability Fact Checking	0.08	0.06	1.44	.150	[-0.03, 0.20]
political interest	-0.01	0.03	-0.40	.692	[-0.06, 0.04]

Note. * p < .05, ** p < .01, *** p < .001

Note: two-tailed regression test with no adjustments made for multiple comparisons

Figure C65: Supplementary Table 38: Skepticism Regression comparing Contextualized and De-Contextualized Strategies in Poland

Term	β	*SE*	*t*	*p*	95% CI
(Intercept)	2.43	0.11	21.53	< .001***	[2.21, 2.66]
age	0.03	0.01	2.30	.021*	[0.00, 0.05]
gender	-0.03	0.04	-0.83	.406	[-0.11, 0.04]
race	0.01	0.01	0.91	.365	[-0.01, 0.04]
education	-0.00	0.01	-0.31	.760	[-0.03, 0.02]
support oppose	-0.00	0.01	-0.33	.744	[-0.01, 0.01]
Control	-0.14	0.04	-3.12	.002**	[-0.23, -0.05]
Correctability Fact Checking	-0.01	0.05	-0.27	.791	[-0.10, 0.08]
political interest	-0.07	0.02	-3.45	.001***	[-0.12, -0.03]

Note. * p < .05, ** p < .01, *** p < .001

Note: two-tailed regression test with no adjustments made for multiple comparisons

Figure C66: Supplementary Table 39: Trust Regression comparing Contextualized and De-Contextualized Strategies in Poland

Term	β	*SE*	*t*	*p*	95% CI
(Intercept)	2.60	0.17	14.84	< .001***	[2.25, 2.94]
age	-0.02	0.02	-1.29	.197	[-0.06, 0.01]
gender	0.13	0.06	2.22	.026*	[0.02, 0.25]
race	0.01	0.02	0.57	.567	[-0.03, 0.05]
education	-0.02	0.02	-0.85	.393	[-0.05, 0.02]
support oppose	0.06	0.01	6.50	< .001***	[0.04, 0.08]
Control	0.07	0.07	1.04	.301	[-0.06, 0.21]
Correctability Fact Checking	0.03	0.07	0.37	.709	[-0.11, 0.17]
political interest	0.24	0.03	7.29	< .001***	[0.18, 0.31]

Note. * p < .05, ** p < .01, *** p < .001

Note: two-tailed regression test with no adjustments made for multiple comparisons

C.1.3 Main Results - Hong Kong

Figure C67: Supplementary Table 40: Misperception by Treatment Regression in Hong Kong

Term	β	*SE*	*t*	*p*	95% CI
(Intercept)	2.70	0.12	21.68	< .001***	[2.46, 2.95]
age	0.01	0.01	0.91	.361	[-0.01, 0.04]
gender	0.06	0.03	2.00	.046*	[0.00, 0.11]
residency	-0.39	0.11	-3.47	.001***	[-0.61, -0.17]
education	-0.01	0.01	-0.93	.351	[-0.03, 0.01]
party	-0.07	0.02	-4.63	< .001***	[-0.10, -0.04]
birth place	0.02	0.06	0.28	.778	[-0.11, 0.14]
Contextualized Media Coverage	-0.04	0.06	-0.62	.533	[-0.15, 0.08]
De-Contextualized Media Coverage	-0.05	0.06	-0.87	.382	[-0.17, 0.06]
Accountability Fact Checking	-0.09	0.06	-1.50	.133	[-0.20, 0.03]
Correctability Fact Checking	-0.09	0.06	-1.63	.104	[-0.21, 0.02]
Bias News Media Literacy	-0.09	0.06	-1.49	.137	[-0.20, 0.03]
Fake News Media Literacy	-0.07	0.06	-1.31	.189	[-0.19, 0.04]
political interest	0.06	0.01	4.47	< .001***	[0.03, 0.09]

Note. * p < .05, ** p < .01, *** p < .001

Note: two-tailed regression test with no adjustments made for multiple comparisons

Figure C68: Supplementary Table 41: Skepticism by Treatment Regression in Hong Kong

Term	β	*SE*	*t*	*p*	95% CI
(Intercept)	2.51	0.10	25.66	< .001***	[2.32, 2.70]
age	-0.01	0.01	-1.23	.218	[-0.03, 0.01]
gender	-0.01	0.02	-0.49	.621	[-0.05, 0.03]
residency	0.03	0.09	0.32	.749	[-0.14, 0.20]
education	0.00	0.01	0.12	.908	[-0.01, 0.01]
party	-0.02	0.01	-1.78	.075	[-0.04, 0.00]
birth place	-0.01	0.05	-0.22	.824	[-0.11, 0.09]
Contextualized Media Coverage	0.01	0.05	0.15	.880	[-0.08, 0.10]
De-Contextualized Media Coverage	0.01	0.05	0.19	.850	[-0.08, 0.10]
Accountability Fact Checking	0.05	0.05	1.00	.317	[-0.04, 0.13]
Correctability Fact Checking	0.03	0.05	0.60	.546	[-0.06, 0.12]
Bias News Media Literacy	0.02	0.05	0.53	.599	[-0.06, 0.11]
Fake News Media Literacy	0.06	0.04	1.29	.198	[-0.03, 0.15]
political interest	-0.07	0.01	-6.35	< .001***	[-0.09, -0.05]

Note. * p < .05, ** p < .01, *** p < .001

Note: two-tailed regression test with no adjustments made for multiple comparisons

Figure C69: Supplementary Table 42: Trust by Treatment Regression in Hong Kong

Term	β	*SE*	*t*	*p*	95% CI
(Intercept)	4.59	0.20	23.50	< .001***	[4.21, 4.98]
age	-0.03	0.02	-1.57	.116	[-0.07, 0.01]
gender	0.07	0.04	1.51	.131	[-0.02, 0.15]
residency	-0.75	0.18	-4.24	< .001***	[-1.09, -0.40]
education	0.01	0.01	0.81	.419	[-0.02, 0.04]
party	0.01	0.02	0.35	.728	[-0.04, 0.05]
birth place	-0.09	0.10	-0.93	.351	[-0.29, 0.10]
Contextualized Media Coverage	0.07	0.09	0.79	.429	[-0.11, 0.25]
De-Contextualized Media Coverage	0.07	0.09	0.76	.449	[-0.11, 0.25]
Accountability Fact Checking	0.00	0.09	0.02	.987	[-0.18, 0.18]
Correctability Fact Checking	0.08	0.09	0.83	.405	[-0.10, 0.26]
Bias News Media Literacy	-0.03	0.09	-0.34	.737	[-0.21, 0.15]
Fake News Media Literacy	0.06	0.09	0.68	.499	[-0.11, 0.24]
political interest	0.14	0.02	6.97	< .001***	[0.10, 0.19]

Note. * p < .05, ** p < .01, *** p < .001

Note: two-tailed regression test with no adjustments made for multiple comparisons

Figure C70: Supplementary Table 43: Trust in Journalists by Treatment Condition in Hong Kong

Term	β	*SE*	*t*	*p*	95% CI
(Intercept)	4.95	0.24	20.75	< .001***	[4.48, 5.42]
age	-0.01	0.02	-0.27	.784	[-0.05, 0.04]
gender	0.06	0.05	1.07	.287	[-0.05, 0.16]
residency	-0.74	0.21	-3.42	.001***	[-1.16, -0.31]
education	-0.01	0.02	-0.74	.462	[-0.05, 0.02]
party	-0.23	0.03	-7.86	< .001***	[-0.28, -0.17]
birth place	-0.02	0.12	-0.16	.872	[-0.26, 0.22]
Contextualized Media Coverage	0.20	0.11	1.78	.076	[-0.02, 0.41]
De-Contextualized Media Coverage	0.15	0.11	1.36	.174	[-0.07, 0.37]
Accountability Fact Checking	0.10	0.11	0.94	.347	[-0.11, 0.32]
Correctability Fact Checking	0.18	0.11	1.63	.103	[-0.04, 0.40]
Bias News Media Literacy	0.06	0.11	0.51	.609	[-0.16, 0.27]
Fake News Media Literacy	0.04	0.11	0.39	.697	[-0.17, 0.26]
political interest	0.18	0.03	7.02	< .001***	[0.13, 0.23]

Note. * p < .05, ** p < .01, *** p < .001

Note: two-tailed regression test with no adjustments made for multiple comparisons

Figure C71: Supplementary Table 44: Trust in Fact Checkers by Treatment Condition in Hong Kong

Term	β	*SE*	*t*	*p*	95% CI
(Intercept)	5.02	0.24	20.82	< .001***	[4.55, 5.49]
age	-0.03	0.02	-1.34	.180	[-0.08, 0.01]
gender	-0.01	0.05	-0.26	.793	[-0.12, 0.09]
residency	-0.56	0.22	-2.58	.010**	[-0.99, -0.13]
education	0.04	0.02	2.42	.015*	[0.01, 0.07]
party	-0.07	0.03	-2.54	.011*	[-0.13, -0.02]
birth place	-0.16	0.12	-1.31	.191	[-0.40, 0.08]
Contextualized Media Coverage	0.08	0.11	0.69	.488	[-0.14, 0.30]
De-Contextualized Media Coverage	0.00	0.11	0.00	.996	[-0.22, 0.22]
Accountability Fact Checking	-0.18	0.11	-1.59	.111	[-0.40, 0.04]
Correctability Fact Checking	-0.03	0.11	-0.30	.761	[-0.26, 0.19]
Bias News Media Literacy	-0.00	0.11	-0.00	.999	[-0.22, 0.22]
Fake News Media Literacy	-0.01	0.11	-0.06	.953	[-0.22, 0.21]
political interest	0.15	0.03	5.86	< .001***	[0.10, 0.20]

Note. * p < .05, ** p < .01, *** p < .001

Note: two-tailed regression test with no adjustments made for multiple comparisons

Figure C72: Supplementary Table 45: Trust in PL Government by Treatment Condition in Hong Kong

Term	β	*SE*	*t*	*p*	95% CI
(Intercept)	3.23	0.33	9.86	< .001***	[2.59, 3.87]
age	0.04	0.03	1.32	.185	[-0.02, 0.11]
gender	-0.00	0.07	-0.04	.968	[-0.15, 0.14]
residency	-1.24	0.29	-4.19	< .001***	[-1.81, -0.66]
education	0.01	0.02	0.27	.785	[-0.04, 0.05]
party	0.51	0.04	12.85	< .001***	[0.43, 0.59]
birth place	-0.13	0.17	-0.78	.433	[-0.46, 0.20]
Contextualized Media Coverage	0.07	0.15	0.47	.639	[-0.23, 0.37]
De-Contextualized Media Coverage	0.07	0.15	0.45	.650	[-0.23, 0.37]
Accountability Fact Checking	0.20	0.15	1.30	.194	[-0.10, 0.49]
Correctability Fact Checking	0.18	0.15	1.15	.251	[-0.12, 0.48]
Bias News Media Literacy	-0.06	0.15	-0.38	.701	[-0.35, 0.24]
Fake News Media Literacy	0.15	0.15	1.01	.314	[-0.14, 0.44]
political interest	0.08	0.03	2.27	.023*	[0.01, 0.15]

Note. * p < .05, ** p < .01, *** p < .001

Note: two-tailed regression test with no adjustments made for multiple comparisons

Figure C73: Supplementary Table 46: Trust in University Professors by Treatment Condition in Hong Kong

Term	β	*SE*	*t*	*p*	95% CI
(Intercept)	4.98	0.24	20.98	< .001***	[4.52, 5.45]
age	-0.10	0.02	-4.22	< .001***	[-0.14, -0.05]
gender	0.05	0.05	0.99	.322	[-0.05, 0.16]
residency	-0.71	0.21	-3.33	.001***	[-1.13, -0.29]
education	0.04	0.02	2.32	.020*	[0.01, 0.07]
party	-0.05	0.03	-1.69	.092	[-0.11, 0.01]
birth place	-0.04	0.12	-0.33	.738	[-0.28, 0.20]
Contextualized Media Coverage	0.06	0.11	0.52	.601	[-0.16, 0.27]
De-Contextualized Media Coverage	0.02	0.11	0.22	.829	[-0.20, 0.24]
Accountability Fact Checking	0.02	0.11	0.22	.829	[-0.19, 0.24]
Correctability Fact Checking	0.07	0.11	0.62	.535	[-0.15, 0.29]
Bias News Media Literacy	-0.05	0.11	-0.45	.652	[-0.27, 0.17]
Fake News Media Literacy	0.12	0.11	1.08	.280	[-0.10, 0.33]
political interest	0.18	0.03	6.95	< .001***	[0.13, 0.22]

Note. * p < .05, ** p < .01, *** p < .001

Note: two-tailed regression test with no adjustments made for multiple comparisons

Figure C74: Supplementary Table 47: Trust in Social Media by Treatment Condition in Hong Kong

Term	β	*SE*	*t*	*p*	95% CI
(Intercept)	4.67	0.25	18.58	< .001***	[4.18, 5.17]
age	-0.05	0.02	-2.21	.027*	[-0.10, -0.01]
gender	0.19	0.06	3.40	.001***	[0.08, 0.30]
residency	-0.76	0.23	-3.36	.001***	[-1.21, -0.32]
education	-0.03	0.02	-1.94	.053	[-0.07, 0.00]
party	-0.14	0.03	-4.63	< .001***	[-0.20, -0.08]
birth place	0.02	0.13	0.14	.885	[-0.23, 0.27]
Contextualized Media Coverage	-0.01	0.12	-0.09	.931	[-0.24, 0.22]
De-Contextualized Media Coverage	-0.05	0.12	-0.40	.690	[-0.28, 0.19]
Accountability Fact Checking	-0.27	0.12	-2.28	.022*	[-0.49, -0.04]
Correctability Fact Checking	-0.03	0.12	-0.27	.789	[-0.26, 0.20]
Bias News Media Literacy	-0.18	0.12	-1.56	.118	[-0.41, 0.05]
Fake News Media Literacy	-0.06	0.11	-0.52	.602	[-0.29, 0.17]
political interest	0.16	0.03	5.92	< .001***	[0.11, 0.21]

Note. * p < .05, ** p < .01, *** p < .001

Note: two-tailed regression test with no adjustments made for multiple comparisons

Figure C75: Supplementary Table 48: Trust in Traditional Media by Treatment Condition in Hong Kong

Term	β	*SE*	*t*	*p*	95% CI
(Intercept)	4.39	0.25	17.29	< .001***	[3.89, 4.89]
age	0.01	0.03	0.48	.634	[-0.04, 0.06]
gender	0.13	0.06	2.25	.024*	[0.02, 0.24]
residency	-0.82	0.23	-3.57	< .001***	[-1.27, -0.37]
education	-0.02	0.02	-1.11	.267	[-0.06, 0.02]
party	0.04	0.03	1.19	.236	[-0.02, 0.10]
birth place	-0.17	0.13	-1.33	.184	[-0.42, 0.08]
Contextualized Media Coverage	0.17	0.12	1.45	.147	[-0.06, 0.40]
De-Contextualized Media Coverage	0.25	0.12	2.07	.039*	[0.01, 0.48]
Accountability Fact Checking	0.13	0.12	1.10	.273	[-0.10, 0.36]
Correctability Fact Checking	0.30	0.12	2.51	.012*	[0.07, 0.53]
Bias News Media Literacy	0.10	0.12	0.82	.415	[-0.13, 0.33]
Fake News Media Literacy	0.18	0.12	1.56	.120	[-0.05, 0.41]
political interest	0.13	0.03	4.64	< .001***	[0.07, 0.18]

Note. * p < .05, ** p < .01, *** p < .001

Note: two-tailed regression test with no adjustments made for multiple comparisons

Figure C76: Supplementary Table 49: Trust in Scientists by Treatment Condition in Hong Kong

Term	β	*SE*	*t*	*p*	95% CI
(Intercept)	4.94	0.23	21.84	< .001***	[4.50, 5.38]
age	-0.04	0.02	-1.92	.055	[-0.09, 0.00]
gender	0.04	0.05	0.80	.422	[-0.06, 0.14]
residency	-0.40	0.20	-1.97	.049*	[-0.80, -0.00]
education	0.04	0.02	2.64	.008**	[0.01, 0.07]
party	0.00	0.03	0.00	1.00	[-0.05, 0.05]
birth place	-0.14	0.11	-1.19	.233	[-0.36, 0.09]
Contextualized Media Coverage	-0.07	0.10	-0.63	.531	[-0.27, 0.14]
De-Contextualized Media Coverage	0.04	0.11	0.36	.716	[-0.17, 0.25]
Accountability Fact Checking	-0.01	0.10	-0.05	.961	[-0.21, 0.20]
Correctability Fact Checking	-0.02	0.11	-0.23	.816	[-0.23, 0.18]
Bias News Media Literacy	-0.08	0.10	-0.77	.441	[-0.29, 0.12]
Fake News Media Literacy	-0.01	0.10	-0.06	.956	[-0.21, 0.20]
political interest	0.14	0.02	5.83	< .001***	[0.09, 0.19]

Note. * p < .05, ** p < .01, *** p < .001

Note: two-tailed regression test with no adjustments made for multiple comparisons

Figure C77: Supplementary Table 50: Misperception Regression comparing Accountability and Correctability Strategies in Hong Kong

Term	β	*SE*	*t*	*p*	95% CI
(Intercept)	2.64	0.19	13.88	< .001***	[2.27, 3.02]
age	0.01	0.02	0.33	.741	[-0.03, 0.04]
gender	0.05	0.04	1.18	.239	[-0.03, 0.14]
residency	-0.31	0.18	-1.77	.077	[-0.66, 0.03]
education	-0.01	0.01	-1.06	.288	[-0.04, 0.01]
party	0.08	0.11	0.77	.443	[-0.13, 0.29]
birth place	-0.10	0.02	-4.04	< .001***	[-0.14, -0.05]
Control	0.09	0.06	1.46	.143	[-0.03, 0.20]
Correctability Fact Checking	-0.01	0.06	-0.19	.851	[-0.12, 0.10]
political interest	0.04	0.02	2.06	.039*	[0.00, 0.09]

Note. * p < .05, ** p < .01, *** p < .001

Note: two-tailed regression test with no adjustments made for multiple comparisons

Figure C78: Supplementary Table 51: Skepticism Regression comparing Accountability and Correctability Strategies in Hong Kong

Term	β	*SE*	*t*	*p*	95% CI
(Intercept)	2.68	0.15	17.96	< .001***	[2.39, 2.97]
age	-0.01	0.02	-0.96	.337	[-0.04, 0.02]
gender	-0.00	0.03	-0.06	.953	[-0.07, 0.06]
residency	-0.12	0.14	-0.87	.383	[-0.39, 0.15]
education	-0.00	0.01	-0.19	.850	[-0.02, 0.02]
party	0.10	0.08	1.23	.219	[-0.06, 0.27]
birth place	-0.03	0.02	-1.57	.116	[-0.07, 0.01]
Control	-0.05	0.05	-1.14	.256	[-0.14, 0.04]
Correctability Fact Checking	-0.02	0.04	-0.53	.594	[-0.11, 0.06]
political interest	-0.08	0.02	-4.98	< .001***	[-0.12, -0.05]

Note. * p < .05, ** p < .01, *** p < .001

Note: two-tailed regression test with no adjustments made for multiple comparisons

Figure C79: Supplementary Table 52: Trust Regression comparing Accountability and Correctability Strategies in Hong Kong

Term	β	*SE*	*t*	*p*	95% CI
(Intercept)	2.68	0.15	17.96	< .001***	[2.39, 2.97]
age	-0.01	0.02	-0.96	.337	[-0.04, 0.02]
gender	-0.00	0.03	-0.06	.953	[-0.07, 0.06]
residency	-0.12	0.14	-0.87	.383	[-0.39, 0.15]
education	-0.00	0.01	-0.19	.850	[-0.02, 0.02]
party	0.10	0.08	1.23	.219	[-0.06, 0.27]
birth place	-0.03	0.02	-1.57	.116	[-0.07, 0.01]
Control	-0.05	0.05	-1.14	.256	[-0.14, 0.04]
Correctability Fact Checking	-0.02	0.04	-0.53	.594	[-0.11, 0.06]
political interest	-0.08	0.02	-4.98	< .001***	[-0.12, -0.05]

Note. * p < .05, ** p < .01, *** p < .001

Note: two-tailed regression test with no adjustments made for multiple comparisons

Figure C80: Supplementary Table 53: Skepticism Regression comparing Bias Focus and Fake News Focus Strategies in Hong Kong

Term	β	*SE*	*t*	*p*	95% CI
(Intercept)	2.68	0.15	17.96	< .001***	[2.39, 2.97]
age	-0.01	0.02	-0.96	.337	[-0.04, 0.02]
gender	-0.00	0.03	-0.06	.953	[-0.07, 0.06]
residency	-0.12	0.14	-0.87	.383	[-0.39, 0.15]
education	-0.00	0.01	-0.19	.850	[-0.02, 0.02]
party	0.10	0.08	1.23	.219	[-0.06, 0.27]
birth place	-0.03	0.02	-1.57	.116	[-0.07, 0.01]
Control	-0.05	0.05	-1.14	.256	[-0.14, 0.04]
Fake News Media Literacy	-0.02	0.04	-0.53	.594	[-0.11, 0.06]
political interest	-0.08	0.02	-4.98	< .001***	[-0.12, -0.05]

Note. * p < .05, ** p < .01, *** p < .001

Note: two-tailed regression test with no adjustments made for multiple comparisons

Figure C81: Supplementary Table 54: Trust Regression comparing Bias Focus and Fake News Focus Strategies in Hong Kong

Term	β	*SE*	*t*	*p*	95% CI
(Intercept)	4.92	0.31	16.10	< .001***	[4.32, 5.52]
age	0.01	0.03	0.48	.635	[-0.05, 0.08]
gender	0.06	0.07	0.82	.411	[-0.08, 0.19]
residency	-0.77	0.28	-2.73	.007**	[-1.33, -0.22]
education	0.00	0.02	0.17	.864	[-0.04, 0.05]
party	-0.36	0.17	-2.12	.034*	[-0.70, -0.03]
birth place	0.03	0.04	0.68	.498	[-0.05, 0.10]
Control	0.00	0.09	0.04	.968	[-0.18, 0.19]
Fake News Media Literacy	0.07	0.09	0.83	.405	[-0.10, 0.25]
political interest	0.09	0.03	2.49	.013*	[0.02, 0.15]

Note. * p < .05, ** p < .01, *** p < .001

Note: two-tailed regression test with no adjustments made for multiple comparisons

Figure C82: Supplementary Table 55: Misperception Regression comparing Contextualized and De-Contextualized Strategies in Hong Kong

Term	β	*SE*	*t*	*p*	95% CI
(Intercept)	2.63	0.19	13.83	< .001***	[2.26, 3.00]
age	0.02	0.02	0.79	.429	[-0.02, 0.05]
gender	0.06	0.04	1.38	.168	[-0.03, 0.14]
residency	-0.50	0.18	-2.80	.005**	[-0.84, -0.15]
education	-0.00	0.01	-0.25	.800	[-0.03, 0.02]
party	-0.06	0.10	-0.57	.569	[-0.26, 0.14]
birth place	-0.05	0.02	-2.21	.027*	[-0.10, -0.01]
Control	0.03	0.06	0.59	.555	[-0.08, 0.15]
De-Contextualized Media Coverage	-0.01	0.06	-0.23	.815	[-0.12, 0.10]
political interest	0.11	0.02	5.21	< .001***	[0.07, 0.15]

Note. * p < .05, ** p < .01, *** p < .001

Note: two-tailed regression test with no adjustments made for multiple comparisons

Figure C83: Supplementary Table 56: Skepticism Regression comparing Contextualized and De-Contextualized Strategies in Hong Kong

Term	β	*SE*	*t*	*p*	95% CI
(Intercept)	2.56	0.15	17.20	< .001***	[2.26, 2.85]
age	-0.02	0.01	-1.02	.306	[-0.04, 0.01]
gender	-0.02	0.03	-0.70	.484	[-0.09, 0.04]
residency	0.05	0.14	0.36	.720	[-0.22, 0.32]
education	0.00	0.01	0.20	.845	[-0.02, 0.02]
party	-0.02	0.08	-0.25	.803	[-0.18, 0.14]
birth place	-0.04	0.02	-2.00	.046*	[-0.08, -0.00]
Control	-0.01	0.05	-0.16	.874	[-0.10, 0.08]
De-Contextualized Media Coverage	0.00	0.04	0.05	.957	[-0.08, 0.09]
political interest	-0.06	0.02	-3.80	< .001***	[-0.09, -0.03]

Note. * p < .05, ** p < .01, *** p < .001

Note: two-tailed regression test with no adjustments made for multiple comparisons

Figure C84: Supplementary Table 57: Trust Regression comparing Contextualized and De-Contextualized Strategies in Hong Kong

Term	β	*SE*	*t*	*p*	95% CI
(Intercept)	4.97	0.29	16.92	< .001***	[4.39, 5.54]
age	-0.04	0.03	-1.36	.174	[-0.10, 0.02]
gender	0.11	0.07	1.68	.094	[-0.02, 0.24]
residency	-1.03	0.27	-3.76	< .001***	[-1.56, -0.49]
education	0.00	0.02	0.09	.927	[-0.04, 0.05]
party	-0.12	0.16	-0.77	.444	[-0.43, 0.19]
birth place	0.00	0.04	0.09	.928	[-0.07, 0.08]
Control	-0.08	0.09	-0.93	.354	[-0.26, 0.09]
De-Contextualized Media Coverage	-0.01	0.09	-0.13	.895	[-0.18, 0.16]
political interest	0.15	0.03	4.81	< .001***	[0.09, 0.22]

Note. * p < .05, ** p < .01, *** p < .001

Note: two-tailed regression test with no adjustments made for multiple comparisons

C.2 Robustness Checks

Figure C85: Supplementary Table 58: United States Compliers Misperception Regression Table

Term	β	*SE*	*t*	*p*	95% CI
(Intercept)	2.98	0.16	18.23	< .001***	[2.65, 3.30]
age	-0.12	0.01	-8.04	< .001***	[-0.15, -0.09]
gender	-0.04	0.05	-0.82	.411	[-0.13, 0.05]
race	-0.03	0.02	-1.21	.227	[-0.07, 0.02]
education	-0.01	0.02	-0.70	.487	[-0.05, 0.03]
party ID	-0.00	0.01	-0.37	.709	[-0.03, 0.02]
Contextualized Media Coverage	-0.23	0.08	-2.90	.004**	[-0.38, -0.07]
De-Contextualized Media Coverage	-0.11	0.10	-1.12	.263	[-0.30, 0.08]
Accountability Fact Checking	-0.39	0.07	-5.64	< .001***	[-0.53, -0.25]
Correctability Fact Checking	-0.46	0.07	-6.54	< .001***	[-0.60, -0.32]
Bias News Media Literacy	-0.02	0.07	-0.28	.782	[-0.16, 0.12]
Fake News Media Literacy	-0.05	0.40	-0.14	.891	[-0.84, 0.73]
political interest	0.05	0.02	2.48	.013*	[0.01, 0.09]

Note. * p < .05, ** p < .01, *** p < .001

Note: two-tailed regression test with no adjustments made for multiple comparisons

Figure C86: Supplementary Table 59: United States Compliers Skepticism Regression Table

Term	β	*SE*	*t*	*p*	95% CI
(Intercept)	2.09	0.12	17.13	< .001***	[1.85, 2.33]
age	0.10	0.01	8.96	< .001***	[0.08, 0.12]
gender	-0.04	0.03	-1.31	.192	[-0.11, 0.02]
race	-0.02	0.02	-1.35	.176	[-0.06, 0.01]
education	0.03	0.02	1.89	.059	[-0.00, 0.06]
party ID	-0.00	0.01	-0.55	.584	[-0.02, 0.01]
Contextualized Media Coverage	0.11	0.06	1.81	.071	[-0.01, 0.22]
De-Contextualized Media Coverage	0.18	0.07	2.56	.011*	[0.04, 0.32]
Accountability Fact Checking	0.07	0.05	1.31	.191	[-0.03, 0.17]
Correctability Fact Checking	0.10	0.05	1.99	.047*	[0.00, 0.21]
Bias News Media Literacy	0.12	0.05	2.28	.023*	[0.02, 0.22]
Fake News Media Literacy	0.10	0.30	0.32	.747	[-0.49, 0.68]
political interest	-0.09	0.01	-6.22	< .001***	[-0.12, -0.06]

Note. * p < .05, ** p < .01, *** p < .001

Note: two-tailed regression test with no adjustments made for multiple comparisons

Figure C87: Supplementary Table 60: United States Compliers Trust Regression Table

Term	β	*SE*	*t*	*p*	95% CI
(Intercept)	4.66	0.23	20.26	< .001***	[4.21, 5.12]
age	-0.09	0.02	-4.24	< .001***	[-0.13, -0.05]
gender	-0.12	0.06	-1.94	.053	[-0.25, 0.00]
race	-0.00	0.03	-0.07	.942	[-0.07, 0.06]
education	0.09	0.03	3.03	.002**	[0.03, 0.14]
party ID	-0.28	0.02	-17.84	< .001***	[-0.31, -0.25]
Contextualized Media Coverage	0.05	0.11	0.43	.665	[-0.17, 0.26]
De-Contextualized Media Coverage	-0.04	0.14	-0.31	.756	[-0.31, 0.22]
Accountability Fact Checking	0.23	0.10	2.33	.020*	[0.04, 0.42]
Correctability Fact Checking	-0.11	0.10	-1.08	.280	[-0.30, 0.09]
Bias News Media Literacy	-0.04	0.10	-0.38	.702	[-0.23, 0.16]
Fake News Media Literacy	-0.53	0.56	-0.95	.342	[-1.64, 0.57]
political interest	0.15	0.03	5.34	< .001***	[0.10, 0.21]

Note. * p < .05, ** p < .01, *** p < .001

Note: two-tailed regression test with no adjustments made for multiple comparisons

Figure C88: Supplementary Table 61: Poland Compliers Misperception Regression Table

Term	β	*SE*	*t*	*p*	95% CI
(Intercept)	2.53	0.11	22.43	< .001***	[2.31, 2.75]
age	-0.07	0.01	-5.89	< .001***	[-0.09, -0.05]
gender	0.02	0.04	0.66	.512	[-0.05, 0.09]
race	0.02	0.01	1.95	.052	[-0.00, 0.05]
education	-0.02	0.01	-1.38	.168	[-0.04, 0.01]
support oppose	0.00	0.01	0.61	.543	[-0.01, 0.01]
Contextualized Media Coverage	-0.07	0.07	-1.00	.319	[-0.20, 0.07]
De-Contextualized Media Coverage	-0.00	0.08	-0.04	.965	[-0.15, 0.15]
Accountability Fact Checking	-0.21	0.06	-3.69	< .001***	[-0.32, -0.10]
Correctability Fact Checking	-0.32	0.05	-6.01	< .001***	[-0.43, -0.22]
Bias News Media Literacy	-0.10	0.06	-1.73	.083	[-0.21, 0.01]
Fake News Media Literacy	-0.00	0.12	-0.03	.977	[-0.23, 0.22]
political interest	-0.08	0.02	-3.75	< .001***	[-0.11, -0.04]

Note. * p < .05, ** p < .01, *** p < .001

Note: two-tailed regression test with no adjustments made for multiple comparisons

Figure C89: Supplementary Table 62: Poland Compliers Skepticism Regression Table

Term	β	*SE*	*t*	*p*	95% CI
(Intercept)	2.16	0.09	23.87	< .001***	[1.98, 2.33]
age	0.01	0.01	1.49	.137	[-0.00, 0.03]
gender	-0.02	0.03	-0.73	.465	[-0.08, 0.04]
race	0.02	0.01	1.78	.076	[-0.00, 0.04]
education	-0.00	0.01	-0.27	.787	[-0.02, 0.02]
support oppose	0.00	0.00	0.41	.680	[-0.01, 0.01]
Contextualized Media Coverage	0.18	0.05	3.40	.001***	[0.08, 0.29]
De-Contextualized Media Coverage	0.15	0.06	2.40	.017*	[0.03, 0.27]
Accountability Fact Checking	0.05	0.05	1.17	.242	[-0.04, 0.14]
Correctability Fact Checking	0.16	0.04	3.72	< .001***	[0.08, 0.24]
Bias News Media Literacy	0.16	0.04	3.69	< .001***	[0.08, 0.25]
Fake News Media Literacy	0.02	0.09	0.20	.843	[-0.16, 0.20]
political interest	-0.03	0.02	-1.75	.080	[-0.06, 0.00]

Note. * p < .05, ** p < .01, *** p < .001

Note: two-tailed regression test with no adjustments made for multiple comparisons

Figure C90: Supplementary Table 63: Poland Compliers Trust Regression Table

Term	β	*SE*	*t*	*p*	95% CI
(Intercept)	2.84	0.14	20.04	< .001***	[2.57, 3.12]
age	-0.02	0.01	-1.06	.287	[-0.04, 0.01]
gender	0.06	0.05	1.42	.156	[-0.02, 0.15]
race	0.02	0.02	1.02	.308	[-0.01, 0.05]
education	-0.00	0.01	-0.17	.861	[-0.03, 0.03]
support oppose	0.07	0.01	9.55	< .001***	[0.06, 0.08]
Contextualized Media Coverage	-0.12	0.09	-1.45	.146	[-0.29, 0.04]
De-Contextualized Media Coverage	-0.16	0.10	-1.64	.102	[-0.35, 0.03]
Accountability Fact Checking	-0.04	0.07	-0.55	.580	[-0.18, 0.10]
Correctability Fact Checking	0.10	0.07	1.45	.148	[-0.03, 0.23]
Bias News Media Literacy	-0.12	0.07	-1.69	.091	[-0.26, 0.02]
Fake News Media Literacy	-0.06	0.15	-0.43	.666	[-0.35, 0.22]
political interest	0.18	0.03	7.07	< .001***	[0.13, 0.23]

Note. * p < .05, ** p < .01, *** p < .001

Note: two-tailed regression test with no adjustments made for multiple comparisons

Figure C91: Supplementary Table 64: United States Wave 2 Misperception Regression

Term	β	*SE*	*t*	*p*	95% CI
(Intercept)	3.25	0.30	10.96	< .001***	[2.67, 3.83]
age	-0.09	0.03	-3.41	.001***	[-0.14, -0.04]
gender	-0.04	0.07	-0.53	.594	[-0.17, 0.10]
race	-0.07	0.04	-1.49	.137	[-0.15, 0.02]
education	-0.01	0.03	-0.40	.691	[-0.07, 0.05]
party ID	0.01	0.02	0.34	.735	[-0.03, 0.04]
Contextualized Media Coverage	-0.16	0.13	-1.28	.201	[-0.41, 0.09]
De-Contextualized Media Coverage	-0.18	0.13	-1.43	.155	[-0.44, 0.07]
Accountability Fact Checking	-0.23	0.12	-1.92	.056	[-0.47, 0.01]
Correctability Fact Checking	-0.08	0.13	-0.68	.500	[-0.33, 0.16]
Bias News Media Literacy	0.07	0.13	0.53	.598	[-0.19, 0.33]
Fake News Media Literacy	-0.09	0.12	-0.73	.469	[-0.33, 0.15]
political interest	-0.02	0.03	-0.56	.578	[-0.08, 0.04]

Note. * p < .05, ** p < .01, *** p < .001

Note: two-tailed regression test with no adjustments made for multiple comparisons

Figure C92: Supplementary Table 65: United States Wave 2 Skepticism Regression

Term	β	*SE*	*t*	*p*	95% CI
(Intercept)	1.83	0.27	6.75	< .001***	[1.30, 2.37]
age	0.08	0.02	3.61	< .001***	[0.04, 0.13]
gender	-0.02	0.06	-0.25	.801	[-0.14, 0.11]
race	-0.02	0.04	-0.41	.678	[-0.10, 0.06]
education	0.03	0.03	0.94	.346	[-0.03, 0.08]
party ID	0.02	0.01	1.05	.296	[-0.01, 0.04]
Contextualized Media Coverage	-0.09	0.12	-0.73	.465	[-0.32, 0.14]
De-Contextualized Media Coverage	0.09	0.12	0.78	.438	[-0.14, 0.32]
Accountability Fact Checking	0.03	0.11	0.29	.774	[-0.19, 0.25]
Correctability Fact Checking	0.05	0.11	0.45	.655	[-0.17, 0.28]
Bias News Media Literacy	-0.02	0.12	-0.15	.880	[-0.26, 0.22]
Fake News Media Literacy	-0.03	0.11	-0.26	.797	[-0.25, 0.19]
political interest	-0.02	0.03	-0.58	.562	[-0.07, 0.04]

Note. * p < .05, ** p < .01, *** p < .001

Note: two-tailed regression test with no adjustments made for multiple comparisons

Figure C93: Supplementary Table 66: United States Wave 2 Trust Regression

Term	β	*SE*	*t*	*p*	95% CI
(Intercept)	4.87	0.49	9.85	< .001***	[3.90, 5.84]
age	-0.09	0.04	-2.01	.045*	[-0.17, -0.00]
gender	0.01	0.11	0.13	.897	[-0.21, 0.23]
race	0.06	0.07	0.80	.423	[-0.09, 0.20]
education	0.03	0.05	0.64	.521	[-0.07, 0.13]
party ID	-0.32	0.03	-11.90	< .001***	[-0.37, -0.26]
Contextualized Media Coverage	-0.31	0.21	-1.44	.152	[-0.72, 0.11]
De-Contextualized Media Coverage	-0.15	0.21	-0.71	.475	[-0.58, 0.27]
Accountability Fact Checking	-0.01	0.20	-0.06	.953	[-0.41, 0.39]
Correctability Fact Checking	-0.32	0.21	-1.52	.130	[-0.73, 0.09]
Bias News Media Literacy	-0.23	0.22	-1.02	.306	[-0.66, 0.21]
Fake News Media Literacy	-0.30	0.21	-1.45	.148	[-0.71, 0.11]
political interest	0.07	0.05	1.40	.163	[-0.03, 0.17]

Note. * p < .05, ** p < .01, *** p < .001

Note: two-tailed regression test with no adjustments made for multiple comparisons

Figure C94: Supplementary Figure 28: Effects of Treatments on US and PL Compliers

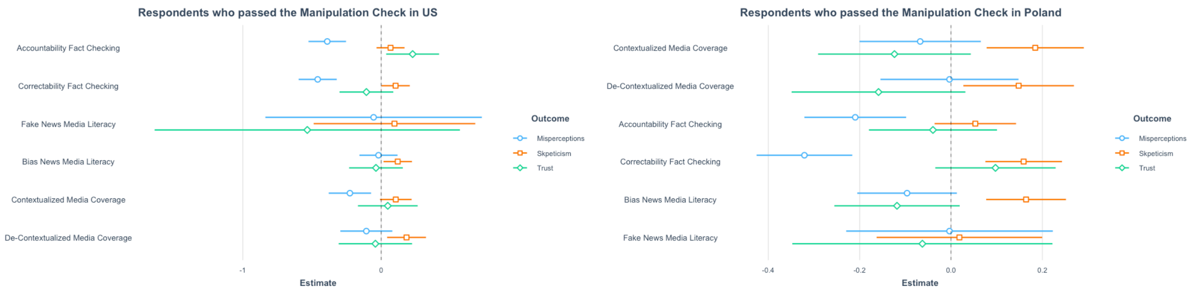

Note: US: n = 2,008 over seven independent treatments, PL: n = 2,147 over seven independent treatments. Data are presented as mean values +/- SD). Two-tailed regression test with no adjustments made for multiple comparisons

Figure C95: Supplementary Figure 29: Effects of Treatments on US Wave 2 Participants

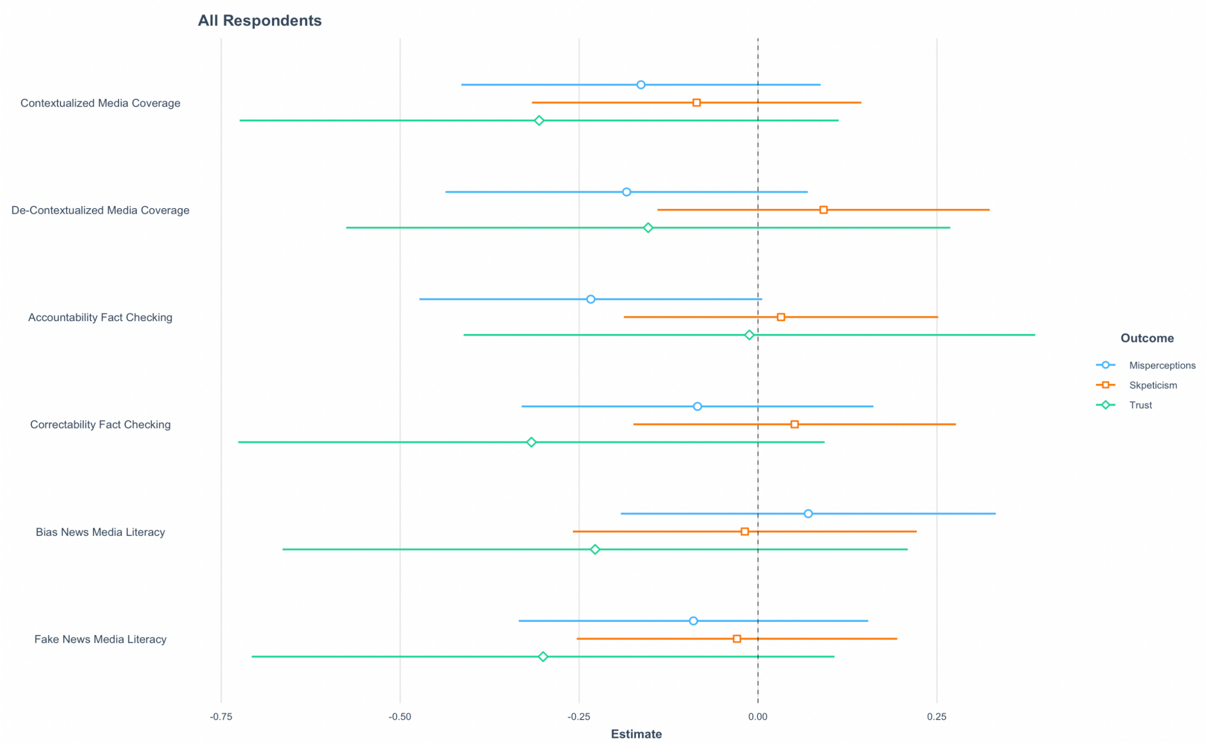

Note: 638 participants over seven independent experiments. Data are presented as mean values +/- SD).Two-tailed regression test with no adjustments made for multiple comparisons.

C.3 Results for other pre-registered outcomes

Figure C96: Supplementary Table 67: United States False Regression

Term	β	*SE*	*t*	*p*	95% CI
(Intercept)	2.15	0.15	14.17	< .001***	[1.85, 2.45]
age	0.10	0.01	7.46	< .001***	[0.07, 0.13]
gender	0.07	0.04	1.66	.098	[-0.01, 0.15]
race	0.03	0.02	1.33	.184	[-0.01, 0.07]
education	-0.03	0.02	-1.77	.077	[-0.07, 0.00]
party ID	0.05	0.01	5.02	< .001***	[0.03, 0.07]
Contextualized Media Coverage	0.05	0.08	0.65	.513	[-0.10, 0.20]
De-Contextualized Media Coverage	-0.03	0.08	-0.40	.688	[-0.18, 0.12]
Accountability Fact Checking	0.03	0.07	0.42	.671	[-0.11, 0.18]
Correctability Fact Checking	0.05	0.08	0.59	.558	[-0.11, 0.20]
Bias News Media Literacy	-0.03	0.08	-0.40	.688	[-0.18, 0.12]
Fake News Media Literacy	-0.03	0.08	-0.45	.650	[-0.19, 0.12]
political interest	-0.03	0.02	-1.60	.110	[-0.06, 0.01]

Note. * p < .05, ** p < .01, *** p < .001

Note: two-tailed regression test with no adjustments made for multiple comparisons

Figure C97: Supplementary Table 68: United States Mainstream Regression

Term	β	*SE*	*t*	*p*	95% CI
(Intercept)	2.83	0.15	18.74	< .001***	[2.53, 3.13]
age	-0.01	0.01	-1.13	.261	[-0.04, 0.01]
gender	-0.01	0.04	-0.21	.831	[-0.09, 0.07]
race	-0.02	0.02	-0.80	.425	[-0.06, 0.02]
education	-0.03	0.02	-1.95	.051	[-0.07, 0.00]
party ID	-0.04	0.01	-4.27	< .001***	[-0.06, -0.02]
Contextualized Media Coverage	-0.02	0.08	-0.22	.829	[-0.17, 0.13]
De-Contextualized Media Coverage	-0.05	0.08	-0.64	.524	[-0.20, 0.10]
Accountability Fact Checking	-0.12	0.07	-1.58	.115	[-0.26, 0.03]
Correctability Fact Checking	-0.05	0.08	-0.64	.522	[-0.20, 0.10]
Bias News Media Literacy	-0.07	0.08	-0.98	.327	[-0.22, 0.07]
Fake News Media Literacy	-0.12	0.08	-1.51	.131	[-0.27, 0.03]
political interest	-0.01	0.02	-0.75	.451	[-0.05, 0.02]

Note. * p < .05, ** p < .01, *** p < .001

Note: two-tailed regression test with no adjustments made for multiple comparisons

Figure C98: Supplementary Table 69: United States Hyperpartisan Regression

Term	β	*SE*	*t*	*p*	95% CI
(Intercept)	2.09	0.14	14.69	< .001***	[1.81, 2.37]
age	0.03	0.01	2.22	.027*	[0.00, 0.05]
gender	-0.06	0.04	-1.46	.144	[-0.13, 0.02]
race	0.10	0.02	4.97	< .001***	[0.06, 0.14]
education	0.07	0.02	4.35	< .001***	[0.04, 0.11]
party ID	-0.02	0.01	-2.56	.010*	[-0.04, -0.01]
Contextualized Media Coverage	0.10	0.07	1.41	.160	[-0.04, 0.24]
De-Contextualized Media Coverage	-0.01	0.07	-0.14	.891	[-0.15, 0.13]
Accountability Fact Checking	0.13	0.07	1.89	.058	[-0.00, 0.27]
Correctability Fact Checking	0.10	0.07	1.37	.171	[-0.04, 0.24]
Bias News Media Literacy	0.15	0.07	2.12	.034*	[0.01, 0.29]
Fake News Media Literacy	0.13	0.07	1.86	.063	[-0.01, 0.28]
political interest	0.05	0.02	2.93	.003**	[0.02, 0.08]

Note. * p < .05, ** p < .01, *** p < .001

Note: two-tailed regression test with no adjustments made for multiple comparisons

Figure C99: Supplementary Table 70: United States Intent to Read Media Lit. Regression

Term	β	*SE*	*t*	*p*	95% CI
(Intercept)	1.38	0.14	10.06	< .001***	[1.11, 1.64]
age	-0.12	0.01	-9.66	< .001***	[-0.14, -0.09]
gender	0.05	0.04	1.31	.191	[-0.02, 0.12]
race	-0.06	0.02	-3.12	.002**	[-0.10, -0.02]
education	0.05	0.02	2.93	.003**	[0.02, 0.08]
party ID	-0.07	0.01	-7.19	< .001***	[-0.08, -0.05]
Contextualized Media Coverage	-0.09	0.07	-1.36	.175	[-0.23, 0.04]
De-Contextualized Media Coverage	-0.19	0.07	-2.83	.005**	[-0.33, -0.06]
Accountability Fact Checking	-0.12	0.07	-1.84	.065	[-0.25, 0.01]
Correctability Fact Checking	-0.09	0.07	-1.29	.197	[-0.23, 0.05]
Bias News Media Literacy	-0.14	0.07	-2.05	.040*	[-0.28, -0.01]
Fake News Media Literacy	-0.09	0.07	-1.33	.185	[-0.23, 0.04]
political interest	0.09	0.02	5.35	< .001***	[0.06, 0.12]

Note. * p < .05, ** p < .01, *** p < .001

Note: two-tailed regression test with no adjustments made for multiple comparisons

Figure C100: Supplementary Table 71: United States Intent to Read Legacy News Regression

Term	β	*SE*	*t*	*p*	95% CI
(Intercept)	1.18	0.14	8.50	< .001***	[0.91, 1.45]
age	0.00	0.01	0.24	.808	[-0.02, 0.03]
gender	0.09	0.04	2.49	.013*	[0.02, 0.17]
race	-0.07	0.02	-3.82	< .001***	[-0.11, -0.04]
education	-0.00	0.02	-0.06	.949	[-0.03, 0.03]
party ID	-0.08	0.01	-8.56	< .001***	[-0.10, -0.06]
Contextualized Media Coverage	-0.06	0.07	-0.85	.393	[-0.20, 0.08]
De-Contextualized Media Coverage	0.02	0.07	0.33	.741	[-0.11, 0.16]
Accountability Fact Checking	0.04	0.07	0.58	.560	[-0.09, 0.17]
Correctability Fact Checking	0.04	0.07	0.57	.568	[-0.10, 0.18]
Bias News Media Literacy	0.02	0.07	0.36	.722	[-0.11, 0.16]
Fake News Media Literacy	0.01	0.07	0.09	.927	[-0.13, 0.14]
political interest	0.12	0.02	7.19	< .001***	[0.09, 0.15]

Note. * p < .05, ** p < .01, *** p < .001

Note: two-tailed regression test with no adjustments made for multiple comparisons

Figure C101: Supplementary Table 72: United States Intent to Read Fake News Regression

Term	β	*SE*	*t*	*p*	95% CI
(Intercept)	0.87	0.13	6.70	< .001***	[0.61, 1.12]
age	-0.09	0.01	-8.05	< .001***	[-0.11, -0.07]
gender	-0.05	0.03	-1.34	.180	[-0.12, 0.02]
race	-0.03	0.02	-1.70	.090	[-0.07, 0.00]
education	0.01	0.02	0.96	.335	[-0.02, 0.04]
party ID	-0.03	0.01	-3.01	.003**	[-0.04, -0.01]
Contextualized Media Coverage	-0.02	0.07	-0.31	.756	[-0.15, 0.11]
De-Contextualized Media Coverage	-0.07	0.07	-1.06	.290	[-0.20, 0.06]
Accountability Fact Checking	-0.01	0.06	-0.17	.869	[-0.13, 0.11]
Correctability Fact Checking	-0.01	0.07	-0.16	.873	[-0.14, 0.12]
Bias News Media Literacy	-0.05	0.07	-0.72	.472	[-0.18, 0.08]
Fake News Media Literacy	-0.00	0.07	-0.03	.975	[-0.13, 0.13]
political interest	0.11	0.02	7.08	< .001***	[0.08, 0.14]

Note. * p < .05, ** p < .01, *** p < .001

Note: two-tailed regression test with no adjustments made for multiple comparisons

Figure C102: Supplementary Table 73: United States Intent to Read Fact Checking Regression

Term	β	*SE*	*t*	*p*	95% CI
(Intercept)	1.03	0.14	7.21	< .001***	[0.75, 1.30]
age	-0.02	0.01	-1.72	.085	[-0.05, 0.00]
gender	0.12	0.04	3.16	.002**	[0.05, 0.20]
race	-0.01	0.02	-0.68	.496	[-0.05, 0.03]
education	0.04	0.02	2.20	.028*	[0.00, 0.07]
party ID	-0.12	0.01	-12.05	< .001***	[-0.13, -0.10]
Contextualized Media Coverage	-0.05	0.07	-0.71	.475	[-0.19, 0.09]
De-Contextualized Media Coverage	-0.02	0.07	-0.32	.752	[-0.16, 0.12]
Accountability Fact Checking	0.01	0.07	0.11	.912	[-0.13, 0.14]
Correctability Fact Checking	-0.04	0.07	-0.59	.558	[-0.19, 0.10]
Bias News Media Literacy	-0.06	0.07	-0.79	.430	[-0.20, 0.08]
Fake News Media Literacy	-0.01	0.07	-0.16	.871	[-0.15, 0.13]
political interest	0.12	0.02	6.83	< .001***	[0.08, 0.15]

Note. * p < .05, ** p < .01, *** p < .001

Note: two-tailed regression test with no adjustments made for multiple comparisons

Figure C103: Supplementary Table 74: Poland False Regression

Term	β	*SE*	*t*	*p*	95% CI
(Intercept)	2.90	0.12	24.69	< .001***	[2.67, 3.14]
age	-0.02	0.01	-1.86	.062	[-0.05, 0.00]
gender	0.02	0.04	0.53	.597	[-0.05, 0.09]
race	-0.00	0.01	-0.16	.871	[-0.03, 0.02]
education	0.01	0.01	0.64	.523	[-0.02, 0.03]
support oppose	0.02	0.01	3.45	.001***	[0.01, 0.03]
Contextualized Media Coverage	-0.02	0.07	-0.34	.736	[-0.16, 0.11]
De-Contextualized Media Coverage	-0.10	0.07	-1.41	.160	[-0.23, 0.04]
Accountability Fact Checking	-0.12	0.07	-1.80	.072	[-0.26, 0.01]
Correctability Fact Checking	-0.01	0.06	-0.13	.893	[-0.13, 0.12]
Bias News Media Literacy	0.09	0.07	1.40	.161	[-0.04, 0.23]
Fake News Media Literacy	0.07	0.07	0.98	.325	[-0.06, 0.19]
political interest	-0.03	0.02	-1.29	.198	[-0.07, 0.01]

Note. * p < .05, ** p < .01, *** p < .001

Note: two-tailed regression test with no adjustments made for multiple comparisons

Figure C104: Supplementary Table 75: Poland Mainstream Regression

Term	β	*SE*	*t*	*p*	95% CI
(Intercept)	2.54	0.13	19.75	< .001***	[2.29, 2.80]
age	0.02	0.01	1.54	.123	[-0.01, 0.05]
gender	-0.07	0.04	-1.76	.079	[-0.15, 0.01]
race	-0.00	0.01	-0.09	.925	[-0.03, 0.03]
education	-0.05	0.01	-4.18	< .001***	[-0.08, -0.03]
support oppose	0.01	0.01	1.80	.073	[-0.00, 0.02]
Contextualized Media Coverage	0.02	0.07	0.21	.832	[-0.13, 0.16]
De-Contextualized Media Coverage	-0.04	0.08	-0.50	.618	[-0.19, 0.11]
Accountability Fact Checking	-0.09	0.07	-1.26	.208	[-0.24, 0.05]
Correctability Fact Checking	-0.08	0.07	-1.20	.232	[-0.22, 0.05]
Bias News Media Literacy	0.01	0.07	0.09	.925	[-0.14, 0.15]
Fake News Media Literacy	-0.11	0.07	-1.51	.132	[-0.25, 0.03]
political interest	-0.05	0.02	-2.23	.026*	[-0.09, -0.01]

Note. * p < .05, ** p < .01, *** p < .001

Note: two-tailed regression test with no adjustments made for multiple comparisons

Supplementary Table 76: Poland Hyperpartisan Regression

Term	β	*SE*	*t*	*p*	95% CI
(Intercept)	2.47	0.13	18.74	< .001***	[2.21, 2.73]
age	-0.04	0.01	-3.19	.001**	[-0.07, -0.02]
gender	0.06	0.04	1.36	.175	[-0.02, 0.14]
race	0.02	0.01	1.37	.170	[-0.01, 0.05]
education	0.06	0.01	4.70	< .001***	[0.04, 0.09]
support oppose	-0.02	0.01	-3.49	< .001***	[-0.04, -0.01]
Contextualized Media Coverage	-0.01	0.08	-0.20	.845	[-0.16, 0.13]
De-Contextualized Media Coverage	0.07	0.08	0.88	.380	[-0.08, 0.22]
Accountability Fact Checking	0.04	0.08	0.54	.590	[-0.11, 0.19]
Correctability Fact Checking	0.02	0.07	0.27	.786	[-0.12, 0.16]
Bias News Media Literacy	0.02	0.08	0.33	.740	[-0.12, 0.17]
Fake News Media Literacy	0.18	0.07	2.48	.013*	[0.04, 0.33]
political interest	0.07	0.02	2.85	.004**	[0.02, 0.11]

Note. * p < .05, ** p < .01, *** p < .001

Note: two-tailed regression test with no adjustments made for multiple comparisons

Figure C105: Supplementary Table 77: Poland Intent to Read Media Lit. Regression

Term	β	*SE*	*t*	*p*	95% CI
(Intercept)	0.42	0.12	3.58	< .001***	[0.19, 0.65]
age	-0.03	0.01	-2.91	.004**	[-0.06, -0.01]
gender	0.09	0.04	2.51	.012*	[0.02, 0.16]
race	0.01	0.01	1.14	.254	[-0.01, 0.04]
education	-0.01	0.01	-0.62	.533	[-0.03, 0.02]
support oppose	0.00	0.01	0.53	.595	[-0.01, 0.01]
Contextualized Media Coverage	-0.08	0.07	-1.13	.258	[-0.21, 0.06]
De-Contextualized Media Coverage	-0.16	0.07	-2.39	.017*	[-0.30, -0.03]
Accountability Fact Checking	-0.11	0.07	-1.69	.090	[-0.25, 0.02]
Correctability Fact Checking	-0.15	0.06	-2.31	.021*	[-0.27, -0.02]
Bias News Media Literacy	-0.21	0.07	-3.13	.002**	[-0.34, -0.08]
Fake News Media Literacy	-0.10	0.07	-1.46	.145	[-0.22, 0.03]
political interest	0.16	0.02	7.74	< .001***	[0.12, 0.20]

Note. * p < .05, ** p < .01, *** p < .001

Note: two-tailed regression test with no adjustments made for multiple comparisons

Figure C106: Supplementary Table 78: Poland Intent to Read Legacy News Regression

Term	β	*SE*	*t*	*p*	95% CI
(Intercept)	0.18	0.11	1.60	.110	[-0.04, 0.41]
age	0.04	0.01	3.06	.002**	[0.01, 0.06]
gender	0.09	0.04	2.47	.013*	[0.02, 0.16]
race	-0.02	0.01	-1.48	.138	[-0.04, 0.01]
education	-0.01	0.01	-1.03	.302	[-0.03, 0.01]
support oppose	0.03	0.01	5.50	< .001***	[0.02, 0.04]
Contextualized Media Coverage	-0.11	0.07	-1.66	.097	[-0.24, 0.02]
De-Contextualized Media Coverage	-0.15	0.07	-2.29	.022*	[-0.28, -0.02]
Accountability Fact Checking	0.03	0.07	0.46	.647	[-0.10, 0.16]
Correctability Fact Checking	-0.10	0.06	-1.55	.120	[-0.22, 0.03]
Bias News Media Literacy	0.05	0.07	0.73	.464	[-0.08, 0.18]
Fake News Media Literacy	-0.06	0.06	-0.94	.348	[-0.19, 0.07]
political interest	0.18	0.02	9.25	< .001***	[0.14, 0.22]

Note. * p < .05, ** p < .01, *** p < .001

Note: two-tailed regression test with no adjustments made for multiple comparisons

Figure C107: Supplementary Table 79: Poland Intent to Read Fake News Regression

Term	β	*SE*	*t*	*p*	95% CI
(Intercept)	0.21	0.11	1.91	.056	[-0.01, 0.43]
age	0.04	0.01	3.98	< .001***	[0.02, 0.07]
gender	0.03	0.03	0.85	.393	[-0.04, 0.10]
race	-0.02	0.01	-1.54	.123	[-0.04, 0.01]
education	-0.03	0.01	-2.62	.009**	[-0.05, -0.01]
support oppose	0.03	0.01	5.19	< .001***	[0.02, 0.04]
Contextualized Media Coverage	-0.04	0.06	-0.58	.565	[-0.16, 0.09]
De-Contextualized Media Coverage	-0.07	0.07	-1.08	.279	[-0.20, 0.06]
Accountability Fact Checking	0.01	0.06	0.17	.864	[-0.12, 0.14]
Correctability Fact Checking	-0.03	0.06	-0.57	.569	[-0.15, 0.08]
Bias News Media Literacy	-0.01	0.06	-0.19	.847	[-0.14, 0.11]
Fake News Media Literacy	-0.00	0.06	-0.03	.973	[-0.13, 0.12]
political interest	0.14	0.02	7.06	< .001***	[0.10, 0.17]

Note. * p < .05, ** p < .01, *** p < .001

Note: two-tailed regression test with no adjustments made for multiple comparisons

Figure C108: Supplementary Table 80: Poland Intent to Read Fact Checking Regression

Term	β	*SE*	*t*	*p*	95% CI
(Intercept)	0.14	0.12	1.18	.238	[-0.09, 0.37]
age	0.01	0.01	0.99	.322	[-0.01, 0.03]
gender	0.11	0.04	2.99	.003**	[0.04, 0.18]
race	0.01	0.01	1.15	.248	[-0.01, 0.04]
education	-0.01	0.01	-0.78	.438	[-0.03, 0.01]
support oppose	-0.01	0.01	-1.79	.074	[-0.02, 0.00]
Contextualized Media Coverage	-0.04	0.07	-0.66	.510	[-0.18, 0.09]
De-Contextualized Media Coverage	-0.11	0.07	-1.61	.108	[-0.24, 0.02]
Accountability Fact Checking	-0.02	0.07	-0.27	.788	[-0.15, 0.11]
Correctability Fact Checking	-0.02	0.06	-0.34	.733	[-0.15, 0.10]
Bias News Media Literacy	0.00	0.07	0.05	.957	[-0.13, 0.13]
Fake News Media Literacy	0.01	0.07	0.10	.917	[-0.12, 0.14]
political interest	0.17	0.02	8.32	< .001***	[0.13, 0.21]

Note. * p < .05, ** p < .01, *** p < .001

Note: two-tailed regression test with no adjustments made for multiple comparisons

Figure C109: Supplementary Table 81: Hong Kong False Regression

Term	β	*SE*	*t*	*p*	95% CI
(Intercept)	2.46	0.15	16.33	< .001***	[2.16, 2.75]
age	0.01	0.01	0.67	.501	[-0.02, 0.04]
gender	-0.05	0.03	-1.61	.108	[-0.12, 0.01]
residency	0.37	0.14	2.75	.006**	[0.11, 0.64]
education	0.01	0.01	1.30	.194	[-0.01, 0.03]
party	-0.02	0.02	-1.36	.175	[-0.06, 0.01]
birth place	-0.02	0.08	-0.30	.768	[-0.17, 0.13]
Contextualized Media Coverage	0.01	0.07	0.15	.877	[-0.13, 0.15]
De-Contextualized Media Coverage	0.04	0.07	0.56	.576	[-0.10, 0.18]
Accountability Fact Checking	0.07	0.07	1.05	.293	[-0.06, 0.21]
Correctability Fact Checking	0.06	0.07	0.84	.403	[-0.08, 0.20]
Bias News Media Literacy	0.12	0.07	1.77	.077	[-0.01, 0.26]
Fake News Media Literacy	0.12	0.07	1.73	.083	[-0.02, 0.25]
political interest	-0.04	0.02	-2.50	.012*	[-0.07, -0.01]

Note. * p < .05, ** p < .01, *** p < .001

Note: two-tailed regression test with no adjustments made for multiple comparisons

Figure C110: Supplementary Table 82: Hong Kong Mainstream Regression

Chunk HTML Output Frame

[illegible]n	β	*SE*	*t*	*p*	95% CI
(Intercept)	2.11	0.14	14.69	< .001***	[1.83, 2.40]
age	-0.00	0.01	-0.09	.929	[-0.03, 0.03]
gender	0.01	0.03	0.26	.798	[-0.05, 0.07]
residency	0.23	0.13	1.74	.081	[-0.03, 0.48]
education	-0.01	0.01	-1.27	.204	[-0.03, 0.01]
party	0.02	0.02	1.08	.282	[-0.02, 0.05]
birth place	-0.06	0.07	-0.80	.423	[-0.20, 0.08]
Contextualized Media Coverage	-0.08	0.07	-1.23	.220	[-0.21, 0.05]
De-Contextualized Media Coverage	0.01	0.07	0.13	.894	[-0.12, 0.14]
Accountability Fact Checking	-0.03	0.07	-0.38	.701	[-0.16, 0.11]
Correctability Fact Checking	-0.07	0.07	-1.01	.314	[-0.20, 0.06]
Bias News Media Literacy	-0.05	0.07	-0.82	.411	[-0.19, 0.08]
Fake News Media Literacy	-0.05	0.07	-0.82	.410	[-0.18, 0.07]
political interest	0.00	0.02	0.30	.765	[-0.03, 0.03]

Note. * p < .05, ** p < .01, *** p < .001

Note: two-tailed regression test with no adjustments made for multiple comparisons

Figure C111: Supplementary Table 83: Hong Kong Hyperpartisan Regression

Term	β	*SE*	*t*	*p*	95% CI
(Intercept)	2.94	0.14	20.56	< .001***	[2.66, 3.22]
age	-0.01	0.01	-1.00	.318	[-0.04, 0.01]
gender	0.00	0.03	0.00	.999	[-0.06, 0.06]
residency	-0.15	0.13	-1.15	.252	[-0.40, 0.10]
education	0.02	0.01	1.87	.062	[-0.00, 0.04]
party	-0.02	0.02	-1.36	.174	[-0.06, 0.01]
birth place	0.02	0.07	0.23	.822	[-0.13, 0.16]
Contextualized Media Coverage	0.02	0.07	0.33	.742	[-0.11, 0.15]
De-Contextualized Media Coverage	-0.05	0.07	-0.69	.490	[-0.18, 0.09]
Accountability Fact Checking	0.01	0.07	0.18	.855	[-0.12, 0.14]
Correctability Fact Checking	-0.05	0.07	-0.68	.500	[-0.18, 0.09]
Bias News Media Literacy	-0.00	0.07	-0.02	.983	[-0.13, 0.13]
Fake News Media Literacy	0.04	0.07	0.67	.501	[-0.08, 0.17]
political interest	-0.00	0.02	-0.04	.970	[-0.03, 0.03]

Note. * p < .05, ** p < .01, *** p < .001

Note: two-tailed regression test with no adjustments made for multiple comparisons

Figure C112: Supplementary Table 84: Hong Kong Intent to Read Media Lit. Regression

Term	β	*SE*	*t*	*p*	95% CI
(Intercept)	0.56	0.34	1.65	.100	[-0.11, 1.23]
age	0.00	0.03	0.15	.883	[-0.05, 0.05]
gender	0.02	0.05	0.33	.739	[-0.09, 0.12]
residency	0.21	0.31	0.67	.504	[-0.40, 0.81]
education	0.01	0.02	0.34	.735	[-0.03, 0.04]
party	0.07	0.03	2.50	.013*	[0.01, 0.12]
birth place	-0.01	0.14	-0.07	.942	[-0.28, 0.26]
Contextualized Media Coverage	-0.08	0.10	-0.77	.442	[-0.28, 0.12]
De-Contextualized Media Coverage	-0.03	0.10	-0.25	.804	[-0.23, 0.18]
Accountability Fact Checking	-0.14	0.10	-1.37	.172	[-0.34, 0.06]
Correctability Fact Checking	-0.10	0.10	-0.99	.321	[-0.30, 0.10]
Bias News Media Literacy	-0.07	0.10	-0.70	.487	[-0.27, 0.13]
Fake News Media Literacy	-0.08	0.10	-0.81	.419	[-0.28, 0.12]
political interest	0.05	0.02	2.10	.036*	[0.00, 0.09]

Note. * p < .05, ** p < .01, *** p < .001

Note: two-tailed regression test with no adjustments made for multiple comparisons

Figure C113: Supplementary Table 85: Hong Kong Intent to Read Legacy News Regression

Term	β	*SE*	*t*	*p*	95% CI
(Intercept)	1.34	0.14	9.25	< .001***	[1.06, 1.63]
age	-0.05	0.01	-3.32	.001***	[-0.08, -0.02]
gender	-0.01	0.03	-0.30	.762	[-0.07, 0.05]
residency	-0.27	0.13	-2.10	.036*	[-0.53, -0.02]
education	0.01	0.01	1.23	.218	[-0.01, 0.03]
party	0.00	0.02	0.13	.897	[-0.03, 0.04]
birth place	-0.20	0.07	-2.69	.007**	[-0.34, -0.05]
Contextualized Media Coverage	0.04	0.07	0.55	.581	[-0.09, 0.17]
De-Contextualized Media Coverage	0.05	0.07	0.79	.428	[-0.08, 0.19]
Accountability Fact Checking	-0.05	0.07	-0.69	.489	[-0.18, 0.08]
Correctability Fact Checking	-0.05	0.07	-0.76	.447	[-0.18, 0.08]
Bias News Media Literacy	-0.01	0.07	-0.14	.892	[-0.14, 0.12]
Fake News Media Literacy	-0.02	0.07	-0.30	.766	[-0.15, 0.11]
political interest	0.10	0.02	6.21	< .001***	[0.07, 0.13]

Note. * p < .05, ** p < .01, *** p < .001

Note: two-tailed regression test with no adjustments made for multiple comparisons

Figure C114: Supplementary Table 86: Hong Kong Intent to Read Fake News Regression

Term	β	*SE*	*t*	*p*	95% CI
(Intercept)	1.23	0.15	8.42	< .001***	[0.95, 1.52]
age	-0.04	0.01	-2.56	.010*	[-0.07, -0.01]
gender	0.06	0.03	1.91	.057	[-0.00, 0.13]
residency	-0.52	0.13	-3.93	< .001***	[-0.78, -0.26]
education	0.00	0.01	0.44	.657	[-0.02, 0.02]
party	-0.04	0.02	-2.44	.015*	[-0.08, -0.01]
birth place	0.14	0.07	1.86	.064	[-0.01, 0.28]
Contextualized Media Coverage	-0.01	0.07	-0.20	.842	[-0.15, 0.12]
De-Contextualized Media Coverage	-0.01	0.07	-0.18	.859	[-0.15, 0.12]
Accountability Fact Checking	0.04	0.07	0.59	.555	[-0.09, 0.17]
Correctability Fact Checking	-0.04	0.07	-0.59	.557	[-0.17, 0.09]
Bias News Media Literacy	-0.07	0.07	-1.04	.297	[-0.20, 0.06]
Fake News Media Literacy	-0.02	0.07	-0.35	.728	[-0.15, 0.11]
political interest	0.09	0.02	5.99	< .001***	[0.06, 0.12]

Note. * p < .05, ** p < .01, *** p < .001

Note: two-tailed regression test with no adjustments made for multiple comparisons

Figure C115: Supplementary Table 87: Hong Kong Intent to Read Fact Checking Regression

Term	β	*SE*	*t*	*p*	95% CI
(Intercept)	1.64	0.15	11.02	< .001***	[1.35, 1.93]
age	-0.03	0.01	-2.25	.024*	[-0.06, -0.00]
gender	0.07	0.03	2.16	.031*	[0.01, 0.14]
residency	-0.30	0.13	-2.21	.027*	[-0.56, -0.03]
education	0.01	0.01	0.74	.460	[-0.01, 0.03]
party	-0.04	0.02	-2.29	.022*	[-0.08, -0.01]
birth place	-0.17	0.08	-2.20	.028*	[-0.32, -0.02]
Contextualized Media Coverage	-0.02	0.07	-0.26	.795	[-0.15, 0.12]
De-Contextualized Media Coverage	0.01	0.07	0.08	.940	[-0.13, 0.14]
Accountability Fact Checking	-0.05	0.07	-0.73	.464	[-0.19, 0.08]
Correctability Fact Checking	-0.15	0.07	-2.10	.036*	[-0.28, -0.01]
Bias News Media Literacy	-0.09	0.07	-1.35	.176	[-0.23, 0.04]
Fake News Media Literacy	-0.02	0.07	-0.24	.807	[-0.15, 0.12]
political interest	0.07	0.02	4.27	< .001***	[0.04, 0.10]

Note. * p < .05, ** p < .01, *** p < .001

Note: two-tailed regression test with no adjustments made for multiple comparisons

C.4 Truth Discernment

Figure C116: Supplementary Table 88: Truth Discernment by Treatment in the US

Term	β	*SE*	*t*	*p*	95% CI
(Intercept)	2.75	0.31	8.96	< .001***	[2.15, 3.36]
age	-0.04	0.03	-1.48	.140	[-0.09, 0.01]
gender	0.25	0.08	3.06	.002**	[0.09, 0.42]
race	0.07	0.04	1.69	.091	[-0.01, 0.15]
education	-0.01	0.04	-0.20	.839	[-0.08, 0.06]
party ID	0.00	0.02	0.20	.840	[-0.04, 0.04]
Contextualized Media Coverage	0.26	0.16	1.67	.094	[-0.04, 0.56]
De-Contextualized Media Coverage	0.00	0.15	0.01	.993	[-0.30, 0.30]
Accountability Fact Checking	0.46	0.15	3.03	.003**	[0.16, 0.75]
Correctability Fact Checking	0.49	0.16	3.15	.002**	[0.19, 0.80]
Bias News Media Literacy	-0.11	0.16	-0.73	.466	[-0.42, 0.19]
Fake News Media Literacy	0.23	0.16	1.46	.143	[-0.08, 0.53]
political interest	0.15	0.04	4.13	< .001***	[0.08, 0.22]

Note. * p < .05, ** p < .01, *** p < .001

Note: two-tailed regression test with no adjustments made for multiple comparisons

Figure C117: Supplementary Table 89: Truth Discernment by Treatment in Poland

Term	β	*SE*	*t*	*p*	95% CI
(Intercept)	3.32	0.26	12.60	< .001***	[2.80, 3.83]
age	0.07	0.03	2.79	.005**	[0.02, 0.13]
gender	0.03	0.08	0.39	.697	[-0.13, 0.19]
race	-0.06	0.03	-2.02	.044*	[-0.11, -0.00]
education	0.09	0.03	3.50	< .001***	[0.04, 0.15]
support oppose	-0.04	0.01	-2.77	.006**	[-0.06, -0.01]
Contextualized Media Coverage	-0.14	0.15	-0.94	.348	[-0.44, 0.16]
De-Contextualized Media Coverage	-0.38	0.15	-2.45	.014*	[-0.68, -0.07]
Accountability Fact Checking	0.26	0.15	1.74	.082	[-0.03, 0.56]
Correctability Fact Checking	0.19	0.14	1.34	.182	[-0.09, 0.47]
Bias News Media Literacy	-0.19	0.15	-1.29	.196	[-0.49, 0.10]
Fake News Media Literacy	0.07	0.15	0.48	.632	[-0.22, 0.36]
political interest	0.22	0.05	4.94	< .001***	[0.14, 0.31]

Note. * p < .05, ** p < .01, *** p < .001

Note: two-tailed regression test with no adjustments made for multiple comparisons

Figure C118: Supplementary Table 90: Truth Discernment by Treatment in Hong Kong

Term	β	*SE*	*t*	*p*	95% CI
(Intercept)	2.10	0.29	7.24	< .001***	[1.53, 2.67]
age	0.03	0.03	1.14	.254	[-0.02, 0.09]
gender	-0.05	0.06	-0.75	.456	[-0.17, 0.08]
residency	0.69	0.26	2.64	.008**	[0.18, 1.20]
education	0.00	0.02	0.22	.827	[-0.04, 0.04]
party	0.18	0.04	5.08	< .001***	[0.11, 0.25]
birth place	-0.05	0.15	-0.33	.738	[-0.34, 0.24]
Contextualized Media Coverage	0.05	0.13	0.39	.695	[-0.21, 0.31]
De-Contextualized Media Coverage	0.11	0.14	0.84	.401	[-0.15, 0.38]
Accountability Fact Checking	0.09	0.13	0.71	.480	[-0.17, 0.36]
Correctability Fact Checking	0.15	0.14	1.07	.284	[-0.12, 0.41]
Bias News Media Literacy	0.13	0.13	0.99	.324	[-0.13, 0.39]
Fake News Media Literacy	-0.01	0.13	-0.09	.929	[-0.27, 0.25]
political interest	0.09	0.03	2.82	.005**	[0.03, 0.15]

Note. * p < .05, ** p < .01, *** p < .001

Note: two-tailed regression test with no adjustments made for multiple comparisons

C.5 Bayes Factor Analyses

Our decision to conduct a Bayes Factor (BF) analysis was driven by the need to interpret several null findings in the Discussion. This approach provides a more nuanced understanding of the data, allowing us to assess the strength of evidence for the absence of an effect, as opposed to merely failing to detect an effect.

Table C1: Supplementary Table 91: Bayes Factors (BF) for differences between existing and proposed strategies per outcome

Intervention	Misperception BF	Skepticism BF
US Fact-Checking	15.43	0.08
US Coverage	1.04	3.10
US Media Literacy	1.57	0.05
PL Fact-Checking	11.74	17.52
PL Coverage	0.08	4.48
PL Media Literacy	0.04	15.50
HK Fact-checking	0.42	0.09
HK Coverage	0.14	0.08
HK Media Literacy	0.16	0.16
