# Supplementary material for: Prominent misinformation interventions reduce misperceptions but increase scepticism
Source: Nat Hum Behav. 2024 Jun 10;8(8):1545–53. doi: 10.1038/s41562-024-01884-x (PMC11343704; doi:10.1038/s41562-024-01884-x)
Supplement: Supplementary file 2 — Reporting Summary [file 41562_2024_1884_MOESM2_ESM.pdf]

nature portfolio

Corresponding author(s): Emma Hoes

Last updated by author(s): 26-01-2024

Reporting Summary

Nature Portfolio wishes to improve the reproducibility of the work that we publish. This form provides structure for consistency and transparency in reporting. For further information on Nature Portfolio policies, see our Editorial Policies and the Editorial Policy Checklist.

Statistics

For all statistical analyses, confirm that the following items are present in the figure legend, table legend, main text, or Methods section.

n/a	Confirmed	
☐	☒	The exact sample size (*n*) for each experimental group/condition, given as a discrete number and unit of measurement
☐	☒	A statement on whether measurements were taken from distinct samples or whether the same sample was measured repeatedly
☐	☒	The statistical test(s) used AND whether they are one- or two-sided<br>*Only common tests should be described solely by name; describe more complex techniques in the Methods section.*
☐	☒	A description of all covariates tested
☐	☒	A description of any assumptions or corrections, such as tests of normality and adjustment for multiple comparisons
☐	☒	A full description of the statistical parameters including central tendency (e.g. means) or other basic estimates (e.g. regression coefficient) AND variation (e.g. standard deviation) or associated estimates of uncertainty (e.g. confidence intervals)
☒	☐	For null hypothesis testing, the test statistic (e.g. *F*, *t*, *r*) with confidence intervals, effect sizes, degrees of freedom and *P* value noted<br>*Give P values as exact values whenever suitable.*
☐	☐	For Bayesian analysis, information on the choice of priors and Markov chain Monte Carlo settings
☐	☒	For hierarchical and complex designs, identification of the appropriate level for tests and full reporting of outcomes
☐	☒	Estimates of effect sizes (e.g. Cohen's *d*, Pearson's *r*), indicating how they were calculated

Our web collection on statistics for biologists contains articles on many of the points above.

Software and code

Policy information about availability of computer code

Data collection	Participants were recruited using Dynata in the US, Panel Ariadna in Poland, and Qualtrics in Hong Kong. These opinion polling companies used stratification based on census information on Age, Gender and Education Level. We calculated sample size for power analysis using the G*Power software (version 3.1.9.6).
Data analysis	Data was analyzed using the statistical software R (version 2022.12.0+353). The code used to analyze the data can be found at https://osf.io/t3nqe

For manuscripts utilizing custom algorithms or software that are central to the research but not yet described in published literature, software must be made available to editors and reviewers. We strongly encourage code deposition in a community repository (e.g. GitHub). See the Nature Portfolio guidelines for submitting code & software for further information.

Data

Policy information about availability of data

All manuscripts must include a data availability statement. This statement should provide the following information, where applicable:

- Accession codes, unique identifiers, or web links for publicly available datasets
- A description of any restrictions on data availability
- For clinical datasets or third party data, please ensure that the statement adheres to our policy

The replication data, including all (stimulus) materials used in this study, is available at https://osf.io/t3nqe

Human research participants

Policy information about studies involving human research participants and Sex and Gender in Research.

Reporting on sex and gender	We introduced a measure of self-reported gender (male/female/other) as a covariate in all our analyses. It was measured asking: "How do you describe yourself?
Population characteristics	Our US dataset included a total N = 2008 participants; mean age = 45 years, 50.22% female, 70.21% white. Our Poland dataset included a total of N = 2147 participants; mean age = 45.65 years, 35.61% female. Finally, our Hong Kong dataset included a total of N = 1972 participants; mean age = 37.93 years, 43.81% female. In the Hong Kong sample, we only had 9.2% of participates aged 55 and older. This age category was 33.1% in the Poland sample and 31.9% in the US sample. These differences may introduce biases.
Recruitment	Participants were recruited using Dynata in the US, Panel Ariadna in Poland, and Qualtrics in Hong Kong. These opinion polling companies used stratification based on census information on Age, Gender and Education Level. Relying on these polling companies may introduce self-selection bias because individuals who choose to participate in online surveys administered by such companies may differ systematically from the broader population, potentially leading to an unrepresentative sample that does not accurately reflect the attitudes and characteristics of the target populations in the US, Poland, and Hong Kong.
Ethics oversight	This study received IRB approval from the University of California Davis, approval no: 1792005-2

Note that full information on the approval of the study protocol must also be provided in the manuscript.

Field-specific reporting

Please select the one below that is the best fit for your research. If you are not sure, read the appropriate sections before making your selection.

☐ Life sciences ☒ Behavioural & social sciences ☐ Ecological, evolutionary & environmental sciences

For a reference copy of the document with all sections, see nature.com/documents/nr-reporting-summary-flat.pdf

Behavioural & social sciences study design

All studies must disclose on these points even when the disclosure is negative.

Study description	Quantitative Online Survey Experiment
Research sample	Participants were recruited using Dynata in the US, Panel Ariadna in Poland, and Qualtrics in Hong Kong. These opinion polling companies used stratification based on census information on Age, Gender and Education Level, and is representative on these demographic variables. Our US dataset included a total N = 2008 participants; mean age = 45 years, 50.22% female, 70.21% white. Our Poland dataset included a total of N = 2147 participants; mean age = 45.65 years, 35.61% female. Finally, our Hong Kong dataset included a total of N = 1972 participants; mean age = 37.93 years, 43.81% female. We selected Poland, Hong Kong, and the US for this survey experiment as these countries represent diverse cultural and political contexts, thus allowing us to explore the generalizability of interventions against misinformation across different societies.
Sampling strategy	Stratified sample (see above). We used the software program G*Power to conduct a power analysis. Our goal was to obtain .80 power to detect a small effect size of 0.02 at the standard 0.05 alpha error probability. We also use a Bonferroni adjustment by dividing the nominal alpha level, 0.05, by the maximum number of comparisons we could make (e.g., not only between the variations of each type of intervention and the control group, but also between types of interventions, and using all the outcomes for each intervention).
Data collection	All data was collected online by the professional opinion polling companies. Data collection and analyses were not performed blind to condition
Timing	The study in each country was conducted from 8/30/22 to 10/27/22. The follow-up survey in the US was conducted from 9/8/22 to 11/5/22.
Data exclusions	Participants who failed the attention check were replaced by the opinion polling companies in each country. This means we eventually did not have to exclude any data from our analyses.
Non-participation	No participants dropped our of declined participation
Randomization	Each participant was randomly assigned to one of six treatment conditions of a control group

Reporting for specific materials, systems and methods

We require information from authors about some types of materials, experimental systems and methods used in many studies. Here, indicate whether each material, system or method listed is relevant to your study. If you are not sure if a list item applies to your research, read the appropriate section before selecting a response.

Materials & experimental systems

n/a	Involved in the study
☒	☐ Antibodies
☒	☐ Eukaryotic cell lines
☒	☐ Palaeontology and archaeology
☒	☐ Animals and other organisms
☒	☐ Clinical data
☒	☐ Dual use research of concern

Methods

n/a	Involved in the study
☒	☐ ChIP-seq
☒	☐ Flow cytometry
☒	☐ MRI-based neuroimaging
